# Genome-Wide Analysis of *MYB* Gene Family in Chinese Bayberry (*Morella rubra*) and Identification of Members Regulating Flavonoid Biosynthesis

**DOI:** 10.3389/fpls.2021.691384

**Published:** 2021-06-24

**Authors:** Yunlin Cao, Huimin Jia, Mengyun Xing, Rong Jin, Donald Grierson, Zhongshan Gao, Chongde Sun, Kunsong Chen, Changjie Xu, Xian Li

**Affiliations:** ^1^Zhejiang Provincial Key Laboratory of Horticultural Plant Integrative Biology, Zhejiang University, Hangzhou, China; ^2^The State Agriculture Ministry Laboratory of Horticultural Plant Growth, Development and Quality Improvement, Zhejiang University, Hangzhou, China; ^3^Institute of Fruit Science, College of Agriculture and Biotechnology, Zhejiang University, Hangzhou, China; ^4^Agricultural Experiment Station, Zhejiang University, Hangzhou, China; ^5^Plant and Crop Sciences Division, School of Biosciences, University of Nottingham, Loughborough, United Kingdom

**Keywords:** Chinese bayberry, MYB transcription factors, transcriptional regulation, anthocyanins, flavonols, proanthocyanidins, flavonoid biosynthesis

## Abstract

Chinese bayberry (*Morella rubra*), the most economically important fruit tree in the Myricaceae family, is a rich source of natural flavonoids. Recently the Chinese bayberry genome has been sequenced, and this provides an opportunity to investigate the organization and evolutionary characteristics of *MrMYB* genes from a whole genome view. In the present study, we performed the genome-wide analysis of *MYB* genes in Chinese bayberry and identified 174 MrMYB transcription factors (TFs), including 122 R2R3-MYBs, 43 1R-MYBs, two 3R-MYBs, one 4R-MYB, and six atypical MYBs. Collinearity analysis indicated that both syntenic and tandem duplications contributed to expansion of the *MrMYB* gene family. Analysis of transcript levels revealed the distinct expression patterns of different *MrMYB* genes, and those which may play important roles in leaf and flower development. Through phylogenetic analysis and correlation analyses, nine MrMYB TFs were selected as candidates regulating flavonoid biosynthesis. By using dual-luciferase assays, MrMYB12 was shown to trans-activate the *MrFLS1* promoter, and MrMYB39 and MrMYB58a trans-activated the *MrLAR1* promoter. In addition, overexpression of 35S:MrMYB12 caused a significant increase in flavonol contents and induced the expression of *NtCHS*, *NtF3H*, and *NtFLS* in transgenic tobacco leaves and flowers and significantly reduced anthocyanin accumulation, resulting in pale-pink or pure white flowers. This indicates that MrMYB12 redirected the flux away from anthocyanin biosynthesis resulting in higher flavonol content. The present study provides valuable information for understanding the classification, gene and motif structure, evolution and predicted functions of the *MrMYB* gene family and identifies MYBs regulating different aspects of flavonoid biosynthesis in Chinese bayberry.

## Introduction

Transcription factors (TFs) are important regulators of gene expression and are generally composed of at least a DNA-binding domain, nuclear location signal, transactivation domain, and an oligomerization site. The MYB family is widely present in all eukaryotes and is one of the largest TF families in plants. MYB proteins are characterized by a highly conserved MYB DNA-binding domain (Dubos et al., 2010). This domain usually comprises up to four imperfect repeats of 50–53 amino acids, and each repeat forms a helix-turn-helix (HTH) structure that binds to DNA and intercalates into the major groove of target DNA sequences (Jia et al., 2004). Based on number of adjacent repeats, MYB TFs can be divided into four classes: 1R-MYB (MYB-related and R3-MYB), R2R3-MYB, 3R-MYB (R1R2R3-MYB), and 4R-MYB (Dubos et al., 2010).

MYB TF families have been previously characterized in various plants, from lower plants such as *Physcomitrella patens* (Dubos et al., 2010) to horticultural plants, such as Chinese pear (*Pyrus bretschneideri*) (Cao et al., 2016) and ornamental flower, *Primulina swinglei* (Feng et al., 2020a). R2R3-MYB and 1R-MYB are the main classes of the MYB family identified. A total of 126 R2R3-MYBs and 64 1R-MYBs have been identified from Arabidopsis (*Arabidopsis thaliana*) (Chen et al., 2006; Dubos et al., 2010). MYB TFs from Arabidopsis are involved in the regulation of many plant processes, including cell fate and identity (Jakoby et al., 2008), organ development (Millar and Gubler, 2005; Silva-Navas et al., 2016), plant metabolism in response to abiotic and biotic stresses (Mehrtens et al., 2005; Dubos et al., 2010; Zhang et al., 2015a).

Recent research has paid more attention to MYB TFs regulating flavonoid metabolism, particularly those related to nutritional value or fruit quality traits. Numerous studies on the regulation of flavonoid biosynthesis have focused on anthocyanins accumulation during fruit development, and MYB TFs were identified in various plants, such as VvMYBA1 and VvMYBA2 in grape (*Vitis vinifera*) (Kobayashi et al., 2002), MdMYB1 in apple (*Malus domestica*) (Takos et al., 2006), and PpMYB10.1, PpMYB10.2, and PpMYB9 in peach (*Prunus persica*) (Rahim et al., 2014; Zhou et al., 2016). For flavonol biosynthesis, AtMYB12 was first reported as a flavonol-specific regulator (Mehrtens et al., 2005), followed by the identification of VvMYBF1 in grape (Czemmel et al., 2009, 2017), MdMYB22 in apple (Wang et al., 2017), and PpMYB15 and PpMYBF1 in peach (Cao et al., 2019). In addition, R2R3-MYB TFs regulating proanthocyanidin (PA) biosynthesis were reported in grape (Deluc et al., 2006) and apple (Wang et al., 2017). Therefore, different MYB members may play specific roles in different branches of flavonoids biosynthesis.

Chinese bayberry (*Morella rubra*), a subtropical fruit tree native to China, is a rich source of natural flavonoids such as anthocyanins, PAs, and flavonols (Yang et al., 2011; Zhang et al., 2015b). A series of investigations by our group have shown that flavonoid-rich pulp extracts of the fruit have a variety of bioactivities including anti-cancer (Sun et al., 2012a), anti-diabetes (Sun et al., 2012b; Zhang et al., 2016), and antioxidant (Zhang et al., 2015b) effects, among others. Previous studies have identified an R2R3-MYB protein, MrMYB1, which acts as a positive regulator of anthocyanin biosynthesis (Niu et al., 2010; Liu et al., 2013). However, the *MYB* genes related to flavonol and PA biosynthesis in Chinese bayberry have not yet been identified. Recently, the Chinese bayberry genome has been sequenced (Jia et al., 2019), and this platform provides an opportunity to identify the *MYB* gene family in Chinese bayberry and to characterize MYB proteins regulating flavonoid biosynthesis.

A comprehensive genome-wide identification of the Chinese bayberry *MYB* gene family was performed in the present study. A total of 174 MrMYB proteins (MrMYBs) were identified and subsequently comprehensively analyzed by phylogenetics, gene structure, identification of conserved motifs, collinearity and determination of chromosomal location. Furthermore, RNA-seq was carried out to investigate expression patterns of *MrMYB* genes in different tissues and during fruit development and *MrMYB* genes related to flavonoid biosynthesis were identified. The function of candidate MYBs in flavonol biosynthesis was examined by transactivation and transformation experiments.

## Materials and Methods

### Plant Materials

All plant materials, including fruit, young leaves, and flowers of Chinese bayberry (*M. rubra* cv. Biqi, BQ) were harvested from commercial orchards in Xianju County, Zhejiang Province, China. The fruit were collected at 45 (S1), 75 (S2), 80 (S3), and 85 (S4) days after full bloom (DAFB). Fifteen fruits or approximately 15 g other tissues for each replicate were sampled and frozen in liquid nitrogen immediately after being cut into small pieces, and all samples were stored at −80°C. Three biological replicates were used for all samples.

### Identification and Sequence Analysis of the *MrMYB* Gene Family

The Hidden Markov Model (HMM) profile of the MYB DNA-binding domain (PF00249) downloaded from Pfam database^1^ was exploited for the identification of *MYB* genes in the Chinese bayberry genome by using the simple HMM search program of TBtools (Chen et al., 2020). The NCBI Conserved Domain Search^2^ and SMART program^3^ were exploited to test for the presence of the MYB domain. The sequence integrity of MrMYBs were analyzed by performing multiple sequence alignment analysis of all MrMYBs by ClustalW^4^ (Chenna et al., 2003). Some MrMYBs containing incomplete MYB domains were found and their coding sequences were individually cloned into pGEM^®^-T Easy Vectors (Promega, Madison, WI, United States). Primers are listed in Supplementary Table 1. After adjusting the multiple sequence alignments manually, we identified the features of R2 and R3 domain repeats by WebLogo^5^ (Crooks et al., 2004). The isoelectric points and protein molecular weights of MrMYBs were obtained through the ExPASy proteomics server^6^.

### Phylogenetic Analyses and Function Predictions of MrMYBs

The protein sequences of MYB proteins from Chinese bayberry and Arabidopsis were aligned by the ClustalW program and adjusted manually, and the multiple sequence alignments were used for phylogenetic analysis. The phylogenetic tree was constructed by the neighbor-joining method of MEGA 7.0 with 1000 bootstrap replicates (Kumar et al., 2016). For the construction of the phylogenetic trees of R2R3-MYB proteins or other MYB proteins from Chinese bayberry, the same method described above was adopted. The phylogenetic trees of all MrMYBs or 21 selected MrMYBs with 30 functional flavonoid-related MYBs from other plants were constructed by the same method as above. Predictions of the biological functions of some MYB proteins were made, according to the orthology based on the aforementioned phylogenetic tree.

### Gene Structure and Conserved Motif Analysis of the *MrMYB* Gene Family

To conduct the classification, GSDS 2.0^7^ (Hu et al., 2015) was used to illustrate exon-intron organization of the *MrMYB* gene family. Furthermore, the Simple MEME program of TBtools was used for identification of conserved motifs in the 174 MrMYB protein sequences. The optimized parameters of MEME were employed as follows: Mode, AnyNumberOfOccurPerSeq; number of motifs to find, 10; and the optimum width of each motif, 6–60 residues. The MEME results were also visualized by TBtools software (Chen et al., 2020).

### Chromosomal Location and Synteny Analysis of the *MrMYB* Gene Family

*MrMYB* genes were located on Chinese bayberry chromosomes according to their positions given in annotated documents of the Chinese bayberry genome using the MapChart software (Voorrips, 2002). The whole-genome sequences and annotation documents of Chinese bayberry and five other selected Rosids species were downloaded to our local server. Then the data were applied to analyze synteny relationships between each pair of Chinese bayberry chromosomes and used for interspecies synteny analyses of *MYB* genes between Chinese bayberry and the other five species using the One Step MCScanx program of TBtools (Chen et al., 2020). While tandem duplications were identified according to the custom script TD_identification^8^ (Feng et al., 2020b). DnaSP v5.0 software was used to calculate the *Ks* value for tandemly and syntenically duplicated *MrMYB* genes (Librado and Rozas, 2009). The duplication pattern of the *MrMYB* gene family was visualized by the Amaizing Super Circos package of TBtools (Chen et al., 2020). The Dual Synteny Plot package of TBtools was used to exhibit interspecies synteny relationships of orthologous *MYB* genes between Chinese bayberry and the other five Rosid species.

### Gene Expression Analysis Using RNA-seq

Total RNA was extracted according to Jia et al. (2019), and its quality was monitored by gel electrophoresis and A_260_/A_280_. Libraries for high-throughput Illumina strand-specific RNA-seq were prepared as described previously (Jia et al., 2019). The RNA-Seq data can be found with accession number PRJNA714192. The expression level of *MrMYB* genes was calculated as fragments per kilobase of exon model per million mapped fragments (FPKM). Three biological replicates for various samples were prepared. Transcript profiles for *MrMYB* genes were obtained and displayed in TBtools (Chen et al., 2020).

### Flavonoid Analyses by HPLC

Flavonoids were analyzed according to Cao et al. (2019) with some modifications. Sample powder (100 mg) was sonicated in 1 ml extraction solution (50% methanol) for 30 min in the dark. After centrifugation at 13,000 rpm for 15 min, the supernatant was collected for HPLC analysis (e2695 pump, 2998 PDA detector, Waters), coupled to an octadecyl silane (ODS) C18 analytical column (4.6 × 250 mm) operated at 25°C, with an injection volume of 10 μl and flow rate of 1 ml/min. The mobile phase for HPLC consisted of 0.1% (v/v) formic acid in water (eluent A) and acetonitrile: 0.1% formic acid (1:1, v/v) (eluent B). The gradient program was as follows: 0–45 min, 23–50% of B; 45–50 min, 50–100% of B; 50–55 min, 100% of B; 55–56 min, 100–23% of B; 56–60 min, 23% of B. Flavonols, anthocyanins, and PAs were detected at 370, 520, and 280 nm, respectively. Contents of flavonoids were calculated by comparison with commercial standards, including myricetin 3-*O*-rhamnoside, quercetin 3-*O*-rutinoside, quercetin 3-*O*-galactoside, quercetin 3-*O*-glucoside, quercetin 3-*O*-rhamnoside, kaempferol 3-*O*-galactoside, kaempferol 3-*O*-glucoside, cyanidin 3-*O*-glucoside, and epigallocatechin gallate. Kaempferol 3-*O*-rutinoside was quantified as kaempferol 3-*O*-glucoside equivalents, other anthocyanins were quantified as cyanidin 3-*O*-glucoside equivalents, and PAs were quantified as epigallocatechin gallate equivalents. Flavonoids in Chinese bayberry tissues were identified by LC-MS according to Yang et al. (2011) and Zhang et al. (2015b). Total flavonols, anthocyanins and PAs contents were the sum of all detected flavonol glycosides, anthocyanins and PAs respectively.

### Dual-Luciferase Assays

Dual-luciferase transactivation activity of TFs on target promoters was performed according to Cao et al. (2019). The full-length coding sequences of MrMYB candidates were individually cloned into pGreenII0029 62_SK vectors. Primers are listed in Supplementary Table 2. Promoters of *MrDFR1^–1557^*, *MrFLS1^–1705^*, *MrLAR1^–1534^*, and *MrANR1^–1512^* were isolated from ‘BQ’ genomic DNA and cloned individually into pGreen II0800_LUC vectors. Primers are listed in Supplementary Table 3. All constructs were electroporated into *Agrobacterium tumefaciens* GV3101. The bacteria were prepared in infiltration buffer (10 mM MES, 10 mM MgCl_2_, 150 mM acetosyringone, pH 5.6) when the optical density at 600 nm reached approximately 0.75. The culture mixtures of bacteria containing TFs (1 ml) and promoters (100 μl) were infiltrated into leaves of 4-week-old *Nicotiana benthamiana* plants. The luminescence from Firefly luciferase (LUC) and Renilla luciferase (REN) was detected by Dual-Luciferase Reporter Assay System (Promega, Madison, WI, United States) on the third day after infiltration, and six biological replicates were used. The ratios of LUC and REN were expressed as activation or repression of the promoters by the TFs.

### Heterologous Transformation and Overexpression

The full-length coding sequence of *MrMYB12* was cloned into pGreenII0029 62_SK containing the cauliflower mosaic virus 35S promoter and transformed into *A. tumefaciens* GV3101. Tobacco (*Nicotiana tabacum*) transformed plants were regenerated as described by Cao et al. (2019). Kanamycin resistant plants were selected and transplanted to soil. The screening procedure was repeated for T1 generation transgenic lines. Fully extended mature leaves of 3-month-old plants (about 50 days after germination) and full-bloom stage flowers were sampled. Three plants were sampled for each transgenic line. Flavonoids were extracted with 50% methanol, analyzed by HPLC, and identified based on retention times and absorbances according to our previous study for quercetin 3-*O*-rutinoside, kaempferol 3-*O*-rutinoside, and cyanidin 3-*O*-glucoside (Cao et al., 2019).

### Real-Time PCR Analysis

Real-time PCR analysis were performed according to Cao et al. (2019). Total RNA of tobacco samples was extracted by TRIzol Reagent kit (Ambion, Unites States). PCRs were performed on a Bio-Rad CFX96 instrument (Bio-Rad), and *NtEF1*-α was used as the internal control for monitoring the abundance of the mRNA. The gene-specific primers proven by melting curves and product resequencing are described in Supplementary Table 4. Expression of genes was calculated by 2^−Δt^.

## Results

### Identification and Sequence Features of *MYB* Genes in Chinese Bayberry

**FIGURE 1:**
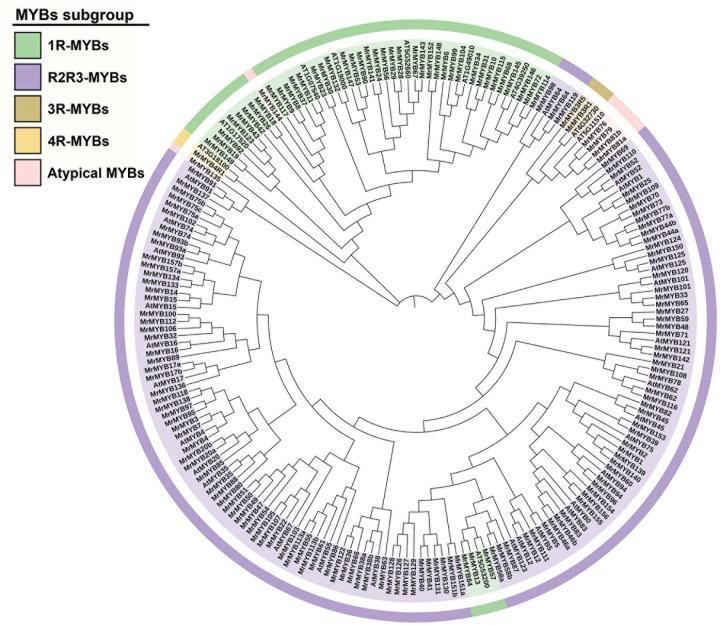
Phylogenetic analysis of MYB proteins from Chinese bayberry and Arabidopsis. A Neighbor-joining phylogenetic tree was constructed by aligning the full-length predicted amino acid sequences of 174 MrMYBs with 37 Arabidopsis MYBs. The classes are shown in different colors.

To identify the *MYB*-encoding genes present in the Chinese bayberry genome, the HMM profile (PF00249) from the Pfam database was used as a query in the HMM search against the genome, and a local BLASTP search was performed by using whole Arabidopsis MYB protein sequences as the query. A total of 276 deduced amino acid sequences that might contain MYB or MYB-like repeats were obtained. All putative *MYB* genes were further examined by the NCBI Conserved Domain Search and SMART program for the presence of the MYB DNA-binding domains. A multiple sequence alignment of all MrMYBs was performed to check the sequence integrity of MrMYBs. Sixteen MrMYBs containing incomplete MYB domains were found, and the sequences of these MrMYBs were corrected through verification of the transcriptome database and cloning and sequence analysis. The updated GenBank numbers of these 16 MrMYBs are provided in Supplementary Table 1. As a result, a total of 174 MYBs were identified in the Chinese bayberry genome. The main sequence information of these MYBs is provided in Supplementary Table 5. A phylogenetic tree of MrMYBs was constructed by aligning the whole set of predicted MYB protein sequences from Chinese bayberry with 37 Arabidopsis MYB protein sequences. As shown in the phylogenetic tree (Figure 1), the MrMYBs were classified into four subfamilies named 1R-MYB (43), R2R3-MYB (122), 3R-MYB (2), and 4R-MYB (1) based on the presence of one, two, three, or four MYB repeats, respectively. Based on the genome data, five MrMYBs contained a complete MYB domain but also contained another incomplete one, and their coding sequences could not be cloned from ‘BQ’ cDNA library for sequence correction. Therefore, these five MYB members are classified as R2R3-MYBs based on the multiple sequence alignment of all MrMYBs.

The MYB domain is the core motif of MYB TFs and is directly involved in binding to the promoters of their target genes. To investigate conservation at specific positions in the MYB domain, sequence logos were generated by the multiple sequence alignment analysis of 122 R2R3-MYBs from Chinese bayberry. As shown in Supplementary Figures 1A,B, the R2 and R3 repeats contain many conserved amino acids, including the characteristic Trp (W) residues, which are recognized landmarks of the MYB domain. Three conserved Trp residues were identified in the R2 repeat. However, only Trp-81 and Trp-100 were conserved in the R3 repeat, and the first Trp at position 62 was substituted with hydrophobic residues, such as Phe (F), Ile (I), or Leu (L), which is a common phenomenon in R2R3-MYB proteins of plants. In addition to the highly conserved Trp residues, Cys-45 and Arg-48 in the R2 repeat, Leu-53 and Pro-55 in the linker region, and Glu-66 and Gly-78 in the R3 repeat were also conserved in the R2R3-MYB proteins.

### The Classification, Motif Composition, and Gene Structure of the *MrMYB* Gene Family

**FIGURE 2:**
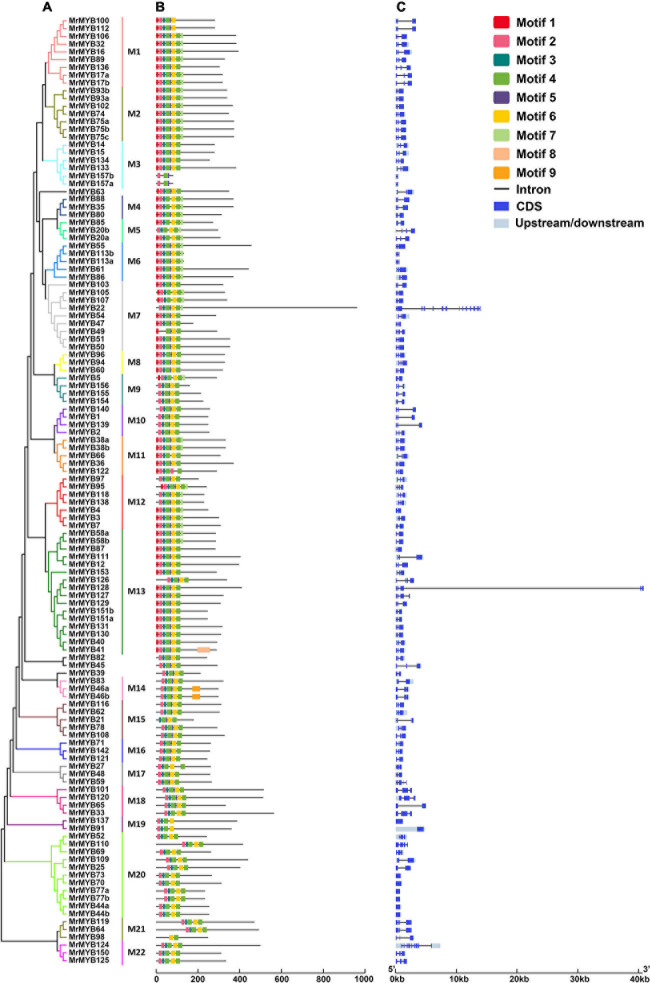
Phylogenetic relationships **(A)**, conserved motifs **(B)**, and gene structure analysis **(C)** of Chinese bayberry *R2R3-MYB* gene family. A Neighbor-joining phylogenetic tree was constructed by aligning the full-length amino acid sequences of 122 R2R3-MYBs in Chinese bayberry. The 22 subgroups are shown with different colors. The blue boxes and black lines in the exon-intron structure diagram represent exons and introns, respectively. The ten conserved motifs are shown in different colors and their specific sequence information is provided in Supplementary Figure 3.

To classify the *MrMYB* genes, two neighbor-joining phylogenetic trees were constructed by using the R2R3-MYB protein sequences or other MYB protein sequences from Chinese bayberry. Based on the support of bootstrap value > 50%, R2R3-MYB proteins from Chinese bayberry could be divided into 22 subgroups (designated M1-M22) (Figure 2A), and the 1R-MYB and 3R-MYB proteins could be divided into seven subgroups (designated I-VII) (Supplementary Figure 2A). Six MrMYBs did not fit into any subgroup, including four R2R3-MYB proteins, one 4R-MYB protein, and one atypical MYB protein.

Subsequently, ten conserved motifs were identified in the MrMYBs through the MEME program (Supplementary Figure 3). The MYB DNA-binding domains were represented by motifs 2, 3, 4, 5, 6, 9, and 10 (Figures 2B and Supplementary Figure 2B). Motif 10 was only present in 1R-MYB TFs, while motifs 1, 5, and 7 only appeared in R2R3-MYB TFs. These results indicated divergence of the MrMYB TFs. Since the analysis of gene structure can help understand the gene function, regulation, and evolution (Feng et al., 2016), the structure of *MrMYB* genes was also examined. As shown in Figure 2C and Supplementary Figure 2C, the number of exons in *MrMYB* genes ranged from one to 15, with an average of 3.6. Among all *MrMYB* genes, 99 *MrMYB* genes contained three exons and accounted for approximately 57% of *MrMYB* gene family members, whereas only 23% of *MrMYB* genes had more than three exons. Most *R2R3-MYB* genes clustered in related groups with similar exon-intron structures, such as M1, M2, M4, M6, etc. (Figure 2C). However, most *1R-MYB*, *3R-MYB*, and atypical *MYB* genes clustered in the same group with different numbers of exons, such as subgroup I-IV, VI, and VII (Supplementary Figure 2C).

### Chromosomal Location and Synteny Analysis of the *MrMYB* Gene Family

**FIGURE 3:**
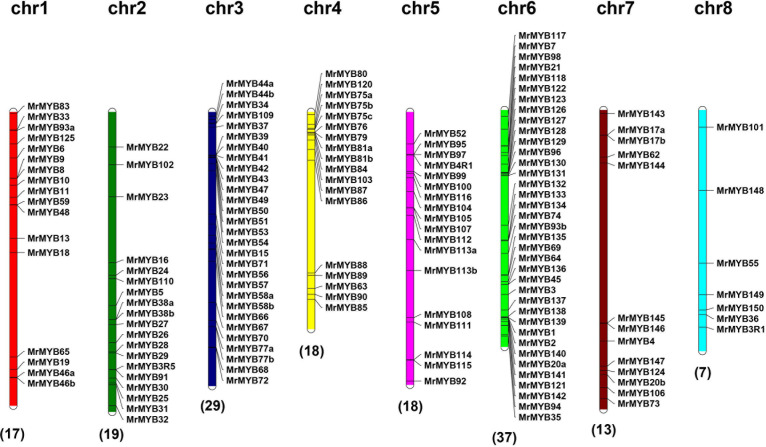
Chromosome distribution of *MrMYB* genes. The chromosome numbers are shown at the top of each chromosome, and the number of *MrMYB* genes in each chromosome is shown at the bottom of each chromosome.

To better understand the genomic distribution of *MrMYB* genes, their positions on each chromosome were marked. This chromosomal location analysis revealed that 158 *MrMYB* genes were unevenly distributed across all eight chromosomes and 16 *MrMYB* genes belonged to unmapped scaffolds (Figure 3). Chromosome 6 had the largest number (37) of *MrMYB* genes, followed by 29 on chromosome 3. In contrast, only seven *MrMYB* genes were found on chromosome 8.

Gene duplication has played a very important role in expansion of gene families (Kent et al., 2003), and high segmental and low tandem duplications were found for the *MYB* gene family in some plants (Cannon et al., 2004; Cao et al., 2016; Liu et al., 2020). We identified a similar number of *MrMYB* tandem duplications (15) and *MrMYB* syntenic duplications (16) in the Chinese bayberry genome (Supplementary Figure 4 and Supplementary Table 6), indicating that both tandem and syntenic duplications contribute to the expansion of *MrMYB* genes because of a lack of recent whole-genome duplication in Chinese bayberry (Jia et al., 2019).

To further explore the evolutionary relationships of *MrMYB* genes with other species, we constructed and compared the syntenic maps of Chinese bayberry with five other Rosid species, including walnut (*Juglans regia*) (Supplementary Figure 5A), Chinese pear (Supplementary Figure 5B), peach (Supplementary Figure 5C), *Medicago truncatula* (Supplementary Figure 5D), and Arabidopsis (Supplementary Figure 5E). A total of 206, 153, 152, 117, and 95 homologous gene pairs were identified between Chinese bayberry and walnut, Chinese pear, peach, *M. truncatula*, and Arabidopsis. These findings likely reflect the closer relationship of Chinese bayberry with walnut, which supported the phylogenetic analysis results of Chinese bayberry and other sequenced species (Jia et al., 2019).

### Expression Pattern of *MrMYB* Genes and in Different Tissues

**FIGURE 4:**
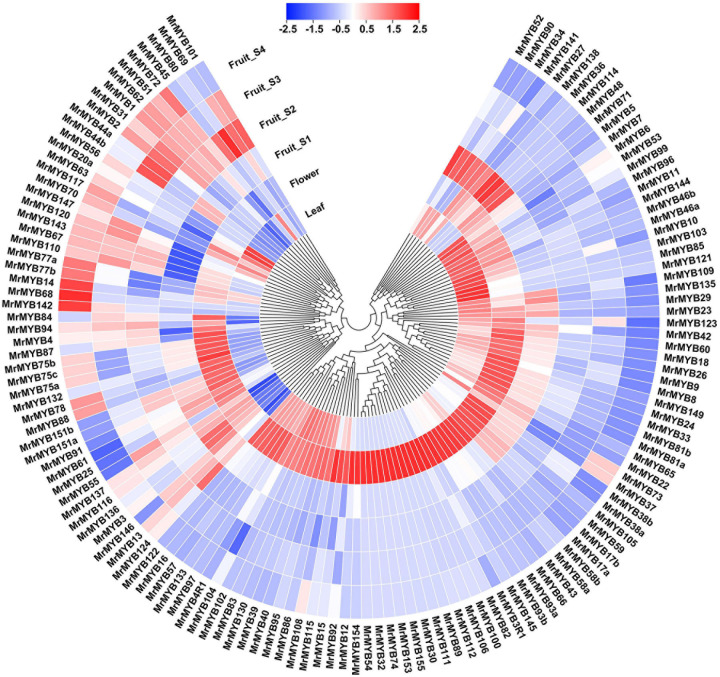
Expression pattern of *MrMYB* genes in different tissues and during fruit development of Chinese bayberry. The expression pattern was generated based on the FPKM after log2 transformation and analyzed by heatmap hierarchical clustering. The color scale (representing −2.5 to 2.5) is shown.

RNA-seq was carried out to examine the expression pattern of 174 *MrMYB* genes in the different tissues, such as leaf, flower, and fruit. The data of transcript levels of *MrMYB* genes is shown in Supplementary Table 7. A total of 79, 38, and 25 *MrMYB* genes showed the highest levels of transcripts in the flower, fruit, and leaf, respectively (Figure 4). Among these *MrMYB* genes showing fruit-specific expression, transcript levels of 14 *MrMYB* genes were the highest at the S1 fruit stage, followed by nine *MrMYB* genes at S2 fruit stage, nine *MrMYB* genes at S3 fruit stage, and six *MrMYB* genes at S4 fruit stage. We also investigated the roles of *MrMYB* genes in regulating fruit development and ripening by analyzing RNA-seq data in the fruit developmental stages. A total of 132 *MrMYB* genes were expressed in the fruit, and 66 of these had an expression level over 1 (FPKM) at any fruit developmental stage and may be involved in regulating fruit development (Figure 4). Of these 132 *MrMYB* genes, transcript levels of 43 *MrMYB* genes were higher at S3 or S4 stage than any other stages, which indicates that these genes may play important roles in regulating aspects of fruit ripening.

### Identification of MrMYBs Regulating Flavonoid Biosynthesis in Chinese Bayberry

**FIGURE 5:**
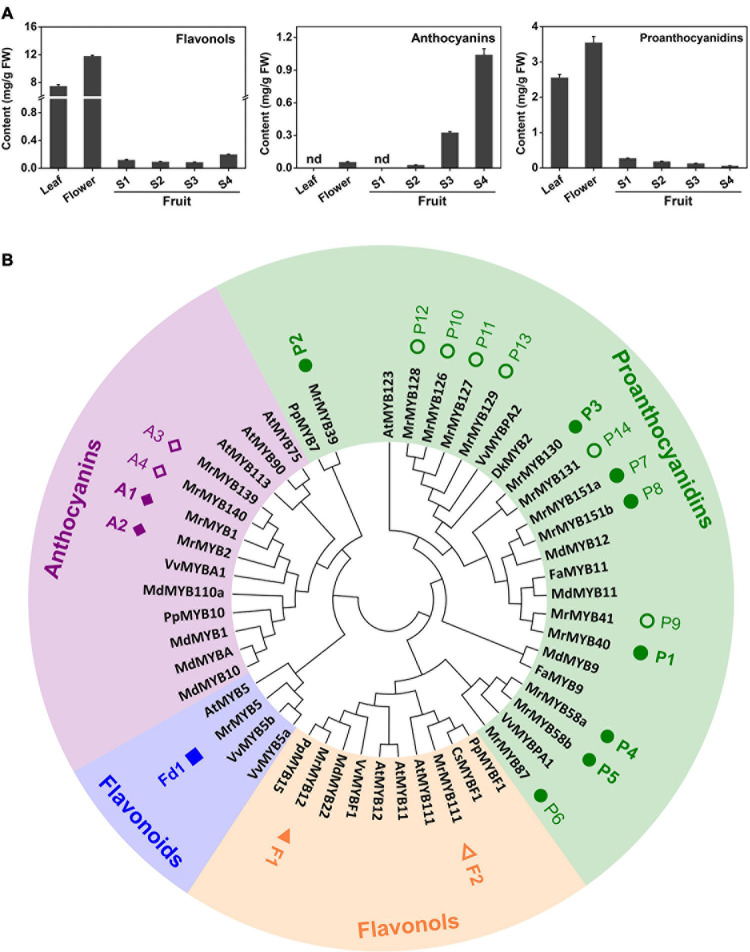
Analysis of Chinese bayberry flavonoid contents **(A)** and phylogeny of MYBs in the anthocyanins, proanthocyanidins, flavonols, and flavonoids clades **(B)**. The square, triangles, rhombuses and circles represent MrMYBs of flavonoids, flavonols, anthocyanins, and proanthocyanidins clades, respectively. The solid symbols indicate the genes expressed in fruit, and the open symbols indicates the genes whose transcripts were not be detected in fruit. Error bars indicate S.E.s from three replicates. FW, fresh weight.

Flavonoid contents in different tissues and during fruit development of Chinese bayberry were analyzed by HPLC. The results indicated that contents of flavonols and PAs were highest in ‘BQ’ flowers, reaching 11.78 and 3.54 mg/g fresh weight (FW), respectively (Figure 5A). Anthocyanins content significantly increased during fruit development and reached the highest level (1.04 mg/g FW) in the mature fruit. In contrast, the PAs level decreased during fruit developmental and flavonols content showed a reducing trend between the S1 and S3 stage but strongly increased at the S4 stage to 0.20 mg/g FW.

To identify additional *MrMYB* genes participating in the regulation of flavonoid biosynthesis in Chinese bayberry, a phylogenetic tree that included all MrMYBs and 30 functional MYBs regulating flavonoid biosynthesis from other species was generated (Supplementary Figure 6). Twenty-one MrMYBs of anthocyanins, flavonols, flavonoids and PAs clades were selected as candidates (Figure 5B and Supplementary Figure 6). We carried out correlation analyses between expression of *MrMYB* genes in the anthocyanins, flavonols, and PAs and flavonoids clades with contents of anthocyanins, flavonols, and PAs respectively. According to the screening criteria (correlation coefficient r > 0.6, *P* < 0.05), two MrMYBs were selected as candidates potentially regulating the biosynthesis of anthocyanins, i.e., MrMYB1 (A1), MrMYB2 (A2), two for flavonols, i.e., MrMYB12 (F1), MrMYB111 (F2), five for PAs, i.e., MrMYB40 (P1), MrMYB39 (P2), MrMYB130 (P3), MrMYB58a/b (P4/P5), and one for flavonoids, i.e., MrMYB5 (Fd1) and used for further screening (Supplementary Table 8). We noted that the sequences of MrMYB58a (P4) and MrMYB58b (P5) had the same coding sequences, and therefore MrMYB58a (P4) was used for further analysis.

**FIGURE 6:**
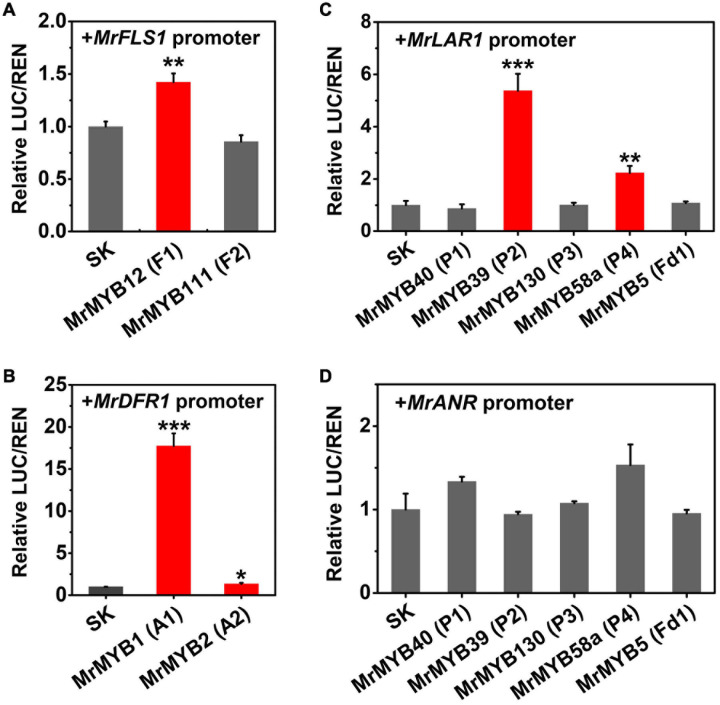
Regulatory effects of flavonoid-related MrMYBs on the transactivation of the promoters of *MrFLS1* **(A)**, *MrDFR1* **(B)**, *MrLAR1* **(C)**, and *MrANR* **(D)**. The ratio of LUC/REN of the empty vector plus promoter was set as 1. SK refers to the empty pGreen II 0029 62-SK vector. Error bars indicate SEs from six replicates (*P < 0.05, **P < 0.01, and ***P < 0.001).

To verify whether the candidate TFs had the ability to regulate flavonoid biosynthesis, dual-luciferase assays were carried out in *N. benthamiana* with potential target genes. It has been well established that DFR, FLS, and LAR and ANR are the key enzymes for anthocyanin, flavonol, and PA biosynthesis, respectively. Therefore, the promoters of these genes were selected as the potential targets of TFs whose transcripts were positively correlated with anthocyanins, flavonols, and PAs accumulation. The results indicated that MrMYB12 (F1) showed a small but significant (1.4-fold) induction of the *MrFLS1* promoter, but MrMYB111 (F2) had no effect (Figure 6A). MrMYB1 (A1) could significantly trans-activate the *MrDFR1* promoter (greater than 17-fold induction), compared to the basal activity set as one (Figure 6B). This verified the previous research on the function of MrMYB1 (Niu et al., 2010; Liu et al., 2013). However, when MrMYB2 (A2) was tested with the same promoter, the transcriptional activity of the *MrDFR1* promoter only increased 1.3 times. With the *MrLAR1* promoter, only MrMYB39 (P2) (5.4-fold) and MrMYB58a (P4) (2.2-fold) could significantly transactivate the transcriptional activity (Figure 6C). However, none of the TFs examined showed significant regulatory effects (neither activation nor repression) on the *MrANR* promoter (Figure 6D).

### Overexpression of *MrMYB12* (F1) Increased Flavonol Accumulation and Reduced Anthocyanin Biosynthesis in Tobacco

**FIGURE 7:**
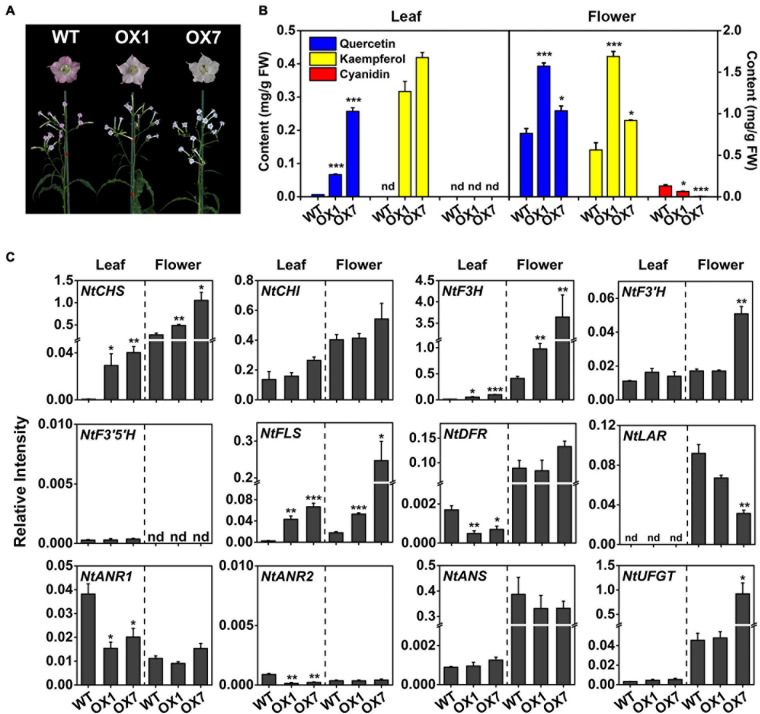
Ectopic expression of *MrMYB12* (F1) genes in tobacco. **(A)** Phenotypes of 35S:MrMYB12 transgenic and wild type (WT) tobacco flowers at the full bloom stage. **(B)** Flavonoid contents of transgenic and WT tobacco flowers and leaves. **(C)** Transcript levels of genes involved in flavonoid biosynthesis in tobacco leaves and flowers overexpressing *MrMYB12* (F1). OX1 and OX7 represent line 1 and line 7 of T1 transgenic plants overexpressing 35S:MrMYB12 (F1), respectively. Student’s *t*-test was used for statistical analyses compared with WT (*P < 0.05, **P < 0.01, and ***P < 0.001). Error bars indicate SEs from three replicates. FW, fresh weight; ND, not detectable.

To confirm that MrMYB12 (F1) functioned as a TF positively regulating flavonol biosynthesis, transgenic tobacco plants were generated overexpressing *MrMYB12* (F1) under the control of the CaMV 35S promoter. Two lines of T1 transgenic plants expressing 35S:MrMYB12 (F1) were used for phenotype analysis. Both transgenic lines accumulated significantly higher levels of quercetin and kaempferol in the leaves and flowers than did the WT, while the anthocyanin content in the flowers represented by the cyanidin content was much lower than WT, consistent with their phenotypic pale-pink or pure white colored flowers (Figures 7A,B). These results indicated that in the transgenic tobacco flowers MrMYB12 (F1) redirected the flux away from anthocyanin biosynthesis, resulting in higher flavonol content. Real-time quantitative PCR analysis showed that overexpression of *MrMYB12* (F1) significantly induced accumulation of *NtCHS*, *NtF3H* and *NtFLS* transcripts (Figure 7C). These results indicated that MrMYB12 (F1) may be a positive regulator of flavonol biosynthesis in Chinese bayberry.

## Discussion

### Identification, Sequence Alignment, and Phylogenetic Analyses of *MrMYB* Gene Family

In the present study, 174 *MrMYB* genes were characterized in Chinese bayberry. This is a higher number of *MrMYB* genes than that in Chinese pear (129) (Cao et al., 2016), and grape (170) (Wilkins et al., 2009; Du et al., 2013) but lower than those in citrus (*Citrus sinensis*) (177) (Hou et al., 2014), Arabidopsis (198) (Chen et al., 2006) and soybean (*Glycine max*) (379) (Du et al., 2012, 2013) (Supplementary Table 9). This indicates that *MYB*s in different plants have expanded to different degrees. It was found that most *MrMYB* genes were not disrupted by more than two introns, which is consistent with previous studies (Du et al., 2012; Liu et al., 2020). The *R2R3-MYB* gene family from Chinese bayberry was classified into 22 subgroups based on phylogenetic analysis, with mostly similar exon-intron organizations and conserved motif compositions. This result is consistent with the previous reports in Arabidopsis, soybean, Chinese pear, and Japanese plum (*Prunus salicina*) (Chen et al., 2006; Du et al., 2012; Cao et al., 2016; Liu et al., 2020), indicating that a strong correlation exists between the phylogenetic topology and gene structures of *R2R3-MYB* genes. However, in our study, all subgroups of the *1R-MYB* gene family in Chinese bayberry consistently displayed a certain degree of divergent in intron-exon organization.

### The *MrMYB* Genes Play Important Roles in Leaf and Flower Development

The combined phylogenetic tree and transcriptomic data analysis provides important information for functional predictions of *MrMYB* genes. Expression analysis by RNA-Seq was conducted in different tissues and during fruit development and ripening in order to investigate the function of *MrMYB* genes. *MrMYB16* was preferentially expressed in flowers and clustered together with AtMYB16 (Figure 1), which contributes to the formation of petal epidermal cells (Baumann et al., 2007), suggesting MrMYB16 may share similar functions in the regulation of petal development. A previous study reported that AtMYB17 may be involved in the regulation of early inflorescence development in Arabidopsis (Zhang et al., 2009), and its homologous genes in Chinese bayberry are *MrMYB17a* and *MrMYB17b*, which are preferentially expressed in the flowers (Figure 4) and thus may have a similar function to AtMYB17. *MrMYB20a/b* and *MrMYB85* were preferentially expressed in leaves (Figure 4) and had a close relationship with AtMYB20 (Figure 1), which participates in regulating lignin and phenylalanine biosynthesis during secondary cell wall formation in Arabidopsis (Geng et al., 2020). This indicates that MrMYB20a/b and MrMYB85 may be involved in the regulation of lignin and phenylalanine biosynthesis in the leaf tissue.

### MrMYB TFs Are Involved in the Regulation of Anthocyanin and PA Biosynthesis in Fruit

Fresh fruits contain a wide range of health-promoting compounds and their regular consumption is one important way to contribute to a healthy diet. Flavonoids are one of the best-accepted health-promoting compounds in fruits and increasing reports have shown that MYB proteins from fruit species are involved in the transcriptional regulation of flavonoid biosynthesis (Falcone Ferreyra et al., 2012; Liu et al., 2015). However, there has been only limited research about the transcriptional regulation of the flavonoid metabolism in Chinese bayberry. Anthocyanins function as pigments and anthocyanin accumulation is one key determinant of fruit color, an important fruit quality attribute. It was found that four MrMYBs were homologous to and clustered with several functional regulators of anthocyanin biosynthesis, such as VvMYBA1 and VvMYBA2 from grape (Kobayashi et al., 2002), MdMYB1 from apple (Takos et al., 2006). Moreover, expression analysis showed that only *MrMYB1* and *MrMYB2* were expressed in any one tissue and transcript levels of these two genes increased with fruit development and ripening, which is consistent with the anthocyanin accumulation pattern in the fruit of Chinese bayberry (Niu et al., 2010; Liu et al., 2013). Dual-luciferase assays in *N. benthamiana* leaf showed that MrMYB1 could significantly trans-activate the *MrDFR1* promoter, which validates the function of MrMYB1 reported by Niu et al. (2010) and Liu et al. (2013). However, MrMYB2 only induced the transcriptional activity 1.3-fold, indicating that MrMYB1 is the more important regulator of anthocyanin biosynthesis in Chinese bayberry. Further study can use controlled crossing breeding materials (Wang et al., 2020).

**FIGURE 8:**
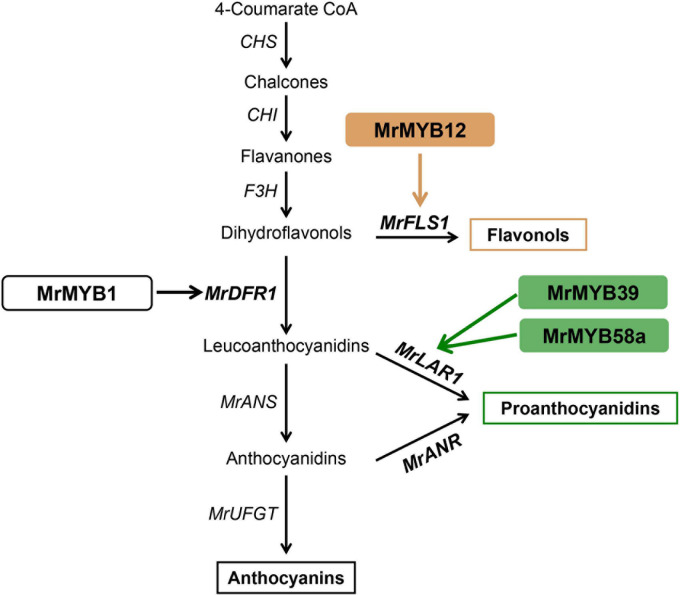
A proposed model for MYB-regulated flavonoid biosynthesis in Chinese bayberry. MrMYB12 activates the expression of *MrFLS1* to regulate flavonol accumulation. The brown and blue arrows indicate the pathways verified in the present work. The black arrows indicate the pathways that have been previously reported in Chinese bayberry. CHS, chalcone synthase; CHI, chalcone isomerase; F3H, flavanone-3-*O*-hydroxylase; DFR, dihydroflavonol-4-reductase; FLS, flavonol synthase; ANS, anthocyanidin synthase; LAR, leucoanthocyanidin reductase; ANR, anthocyanidin reductase; UFGT, UDP-glucose:flavonoid 3-*O*-glucosyltransferase.

PAs are distributed widely in the leaves and fruit of Chinese bayberry and have been associated with health-promoting benefits. A previous study has functionally characterized two key genes of PA biosynthesis, *MrLAR1* and *MrANR*, but the mechanism regulating PA biosynthesis remains unclear. We found (Figure 5B) that 14 MrMYBs were clustered with the PA clade of the MYB family, and MrMYB5 (Fd1) in the flavonoid clade was homologous with VvMYB5a and VvMYB5b which are known to be involved in regulating PA biosynthesis (Deluc et al., 2006, 2008). Among five MrMYBs screened by the correlation analyses, only MrMYB39 (P2) and MrMYB58a (P4) significantly activated the promoter of *MrLAR1* but did not activate that of *MrANR* (Figures 6C,D). Similar results were found in apple MYB12 and peach MYB7, which regulate the biosynthesis of catechin but not epicatechin (Zhou et al., 2015; Wang et al., 2017). Different results were obtained previously with MdMYB9, VvMYBPA1, and VvMYBPA2, which could regulate the expression of *LAR* and *ANR* to promote the accumulation of catechin and epicatechin (Bogs et al., 2007; Terrier et al., 2009; An et al., 2015), and small differences in the amino acid sequences of these proteins may account for this. Therefore, the biosynthesis of catechin and epicatechin may be regulated by different MYB TFs. It is clear that MrMYB39 (P2) and MrMYB58a (P4) may function as positive regulators of flavonoid biosynthesis by regulating the transcription of *MrLAR1* (Figure 8).

### MrMYB12 (F1) Function as A Flavonol-Specific Regulator

Flavonols, a class of colorless flavonoids, are important health-related compounds in the human diet. Previous studies reported that the regulation of flavonol biosynthesis is usually controlled by the SG7 subgroup of the MYB family (Mehrtens et al., 2005; Stracke et al., 2007). Two MrMYBs, MrMYB12 (F1) and MrMYB111 (F2), were clustered in the flavonol clade with the functional flavonol regulators from other plants and expression levels of these two genes were highly correlated with the flavonols content. Dual-luciferase assays *in vivo* indicated that MrMYB12 (F1) trans-activated the *MrFLS1* gene promoter (Figure 6A) as does its homologs, AtMYB12, VvMYBF1, MdMYB22, and PpMYB15 (Mehrtens et al., 2005; Czemmel et al., 2009; Wang et al., 2017; Cao et al., 2019). However, MrMYB111, a homolog of PpMYBF1, failed to activate the *MrFLS1* gene promoter. Previously, over-expression of *AtMYB12* resulted in unprecedentedly high levels of kaempferol or quercetin accumulation in both tobacco and tomato, and lower anthocyanin levels (Luo et al., 2008). Accordingly, the content of kaempferol or quercetin was reduced significantly in *Slmyb12* or *Atmyb12* mutants (Mehrtens et al., 2005; Ballester et al., 2010). Consistent with these reports, our data show that over-expression of 35S:MrMYB12 (F1) in tobacco promoted kaempferol or quercetin accumulation and decreased anthocyanin accumulation by upregulating the transcript levels of *NtCHS*, *NtF3H* and *NtFLS*. Therefore, MrMYB12 (F1) may act as a flavonol-specific regulator by redirecting the flux from anthocyanin biosynthesis to flavonol biosynthesis (Figure 8).

## Conclusion

Genome-wide analysis of phylogenetic relationships, gene structures, motif compositions, chromosomal locations, evolutionary relationships, and expression of *MrMYB* genes, was carried out in the present study. A total of 174 MYB family members from Chinese bayberry were identified. Intraspecies synteny analysis indicated that both dispersed syntenic and tandem duplications contributed to expansion of the *MrMYB* gene family. Expression analysis revealed that *MrMYB* genes had tissue-specific expression patterns in leaf, flower and fruit, and some were identified as likely to have important roles in leaf and flower development, consistent with the functional predictions from phylogenetic analysis. Through the combination of phylogenetic analysis and correlation analyses, nine MrMYB TFs were selected as candidates associated with flavonoid biosynthesis. Of these candidates, MrMYB12 trans-activated the *MrFLS1* promoter, and MrMYB39 and MrMYB58a activated the *MrLAR1* promoter. In addition, heterologous overexpression of 35S:MrMYB12 increased flavonol levels and induced the expression of *NtCHS*, *NtF3H*, and *NtFLS* in transgenic tobacco leaves and flowers and significantly reduced anthocyanin accumulation, resulting in pale-pink or pure white flowers. Overall, these results provide information that will facilitate further functional analyses of *MrMYB* genes to elucidate their biological roles. The functional identification of different MYBs regulating flavonoid biosynthesis will help to improve the fruit quality of Chinese bayberry in the future.

## Data Availability Statement

The original contributions presented in the study are publicly available. This data can be found here: RNA-Seq data can be found with accession number PRJNA714192. The RNA-Seq data is publicly available on National Center for Biotechnology Information.

## Author Contributions

XL and YC designed the project and drafted the manuscript. YC managed the experiments with help from HJ, MX, and RJ. CX and KC participated in design of the study and provided support for the Morella project. CX, DG, CS, and ZG contributed to the discussion and revision of the manuscript. All authors approved the article.

## Conflict of Interest

The authors declare that the research was conducted in the absence of any commercial or financial relationships that could be construed as a potential conflict of interest.

## Footnotes

1 http://pfam.sanger.ac.uk/

2 http://www.ncbi.nlm.nih.gov/Structure/cdd/wrpsb.cgi

3 http://smart.embl-heidelberg.de/

4 http://www.ebi.ac.uk/Tools/msa/clustalw2/

5 http://weblogo.berkeley.edu/logo.cgi

6 http://www.expasy.org/tools/

7 http://gsds.gao-lab.org/index.php

8 https://github.com/scbgfengchao/

## Supplementary Material

Supplementary Figure 1 The sequence logos of the R2 **(A)** and R3 **(B)** MYB repeats. These logos were based on the multiple sequence alignment of 122 R2R3-MYBs in Chinese bayberry. The core bits indicate the information content for each position in the sequence. The asterisks indicate the typical conserved Trp residues in the MYB domain. The triangle indicates the residue in the R3 repeat with positions identical to the first conserved Trp residue in the R2 repeat.

Supplementary Figure 2 Phylogenetic relationships **(A)**, conserved motifs **(B)**, and gene structure analysis **(C)** in Chinese bayberry 1R-, 3R-, and 4R-MYBs. A Neighbor-joining phylogenetic tree was constructed by aligning the full-length amino acid sequences of 1R-, 3R-, and 4R-MYBs. The seven subgroups are shown in different colors. The blue boxes and black lines in the exon-intron structure diagram represent exons and introns, respectively. The ten conserved motifs are exhibited with different colors and their specific sequence information is provided in Supplementary Figure 3.

Supplementary Figure 3 All MEME motif sequence logos in MrMYBs.

Supplementary Figure 4 Schematic representations for the interchromosomal relationships of *MrMYB* genes. **(A)** Gray lines in the background mean collinear blocks within Chinese bayberry, red lines indicate syntenic *MYB* gene pairs. **(B)** Duplicated gene pairs of *MrMYB* genes are sorted according to their assigned MYB classes.

Supplementary Figure 5 Syntenic analysis of *MYB* genes between Chinese bayberry and *Juglans regia* **(A)**, *Pyrus bretschneideri* **(B)**, *Prunus persica* **(C)**, *Medicago truncatula* **(D)**, or *Arabidopsis thaliana* **(E)**. Gray lines in the background mean collinear blocks between Chinese bayberry and other plant genomes, and red lines indicate syntenic *MYB* gene pairs.

Supplementary Figure 6 Phylogenetic analysis of all MrMYBs and functional flavonoid-related MYB proteins from other plants. The clades are shown in different colors.

Supplementary Table 1 Primers for *MrMYB* gene amplification and T-easy vector constructions.

Supplementary Table 2 Primers for promoter amplification and pGreenII0029 62_SK vector constructions. Sequences of restriction sites are underlined.

Supplementary Table 3 Primers for promoter amplification and pGreen II0800_LUC vector constructions. Sequences of restriction sites are underlined.

Supplementary Table 4 Primers used for quantitative real-time PCR analysis.

Supplementary Table 5 The isoelectric point, molecular weight, chromosome location, and MYB-domain type of the members of *MrMYB* gene family. The principles of gene naming are as follows: (1) Gene names of the *MrMYB* locus have been reported; (2) *MrMYB* genes were named based on their homologous genes in Arabidopsis; (3) without meeting the first and second principles, *MrMYB* genes were named according to the order of chromosome location.

Supplementary Table 6 The *Ks* value for tandemly and syntenically duplicated *MrMYB* genes.

Supplementary Table 7 Transcript levels of *MrMYB* genes in different tissues and during fruit development. L, leaf; Fl, flower; Fr, fruit.

Supplementary Table 8 Correlation between flavonoid-related *MrMYB* genes with flavonols, anthocyanins or proanthocyanidins contents. Expression patterns of the *MrMYB* genes and flavonoid profiles in different tissues and during fruit development are shown in Figures 4, 5.

Supplementary Table 9 Numbers of plant MYBs in the four different classes.
