# Supplementary material for: Genome-Wide Analysis of MYB Gene Family in Chinese Bayberry (Morella rubra) and Identification of Members Regulating Flavonoid Biosynthesis
Source: Front Plant Sci. 2021 Jun 24;12:691384. doi: 10.3389/fpls.2021.691384 (PMC8264421; doi:10.3389/fpls.2021.691384)

**Table S1** Primers for *MrMYB* gene amplification and T-easy vector constructions.

| **Gene name** | **Accession number** | **Updated number** | **Forward primers** | **Reverse primers** |
| --- | --- | --- | --- | --- |
| *MrMYB154* | KAB1200773.1 | MW835212 | ATGGAAGCAGAGAATC | TCAATAAAAGGAAGAGGA |
| *MrMYB55* | KAB1203457.1 | MW835213 | ATGGGAAGGCACTCTTG | CTAAGGATGTCCAAATGCCG |
| *MrMYB3R1* | KAB1204398.1 | MW835214 | ATGATTTGGAGAGCTTATTCTGGAG | AGCCACAGCCATAGGAAACC |
| *MrMYB106* | KAB1208247.1 | MW835215 | TCAACCATGGGCAGGTCTCC | TCAGAACACAGGCGAACC |
| *MrMYB21* | KAB1209160.1 | MW835216 | ATGGATAAGAAACCATGCAACT | TTAATCCCCACTAAATAGATGC |
| *MrMYB136* | KAB1210709.1 | MW835217 | ATGGGAAGGGCACCATGT | TTAGTAACATTTTGTGTCTAGCAA |
| *MrMYB139* | KAB1211294.1 | MW835218 | ATGGAAGGCTCTTTAGGTG | CCGTTATTATGGATCGAGAA |
| *MrMYB1* | KAB1211297.1 | ADG21957 | ATGGAAGGCTCTTTAGGTGTAC | TTATGGATCGAGAAAATCCCAAAC |
| *MrMYB2* | KAB1211298.1 | MW835219 | ATGGAGCGCTCCTCCAGTGTG | TTAGATCAGCGCTCCTTCTTCG |
| *MrMYB140* | KAB1211299.1 | MW835220 | ATGTCTCAACGTTCGGG | TTATGGATCGAGAAAATCCCAAACG |
| *MrMYB105* | KAB1212833.1 | MW835221 | ATGTTAAACTCTAAGATTGGCG | TCAAATCAGAATACATAAAACCA |
| *MrMYB107* | KAB1212835.1 | MW835222 | ATGTTAAACGCTAAGATTGG | CTAGTTCCATGCAGTATCAT |
| *MrMYB103* | KAB1215385.1 | MW835223 | ATGGGTCACCATTCTTGC | TTATAGTGACGAAGGGAAAGC |
| *MrMYB87* | KAB1215514.1 | MW835224 | ATGGGAAGAGCCCCATGCTGT | TCAAATAAGCAAGGAATCGACAAAGG |
| *MrMYB25* | KAB1224278.1 | MW835225 | ATGGAAGTGGAGGAC | CTAGGTTGACAGAACATTC |
| *MrMYB33* | KAB1225022.1 | MW835226 | ATGAAACGCAAAACAACTGA | TCAAGGAAGCCCAGACATCT |

**Table S2** Primers for promoter amplification and pGreenII0029 62_SK vector constructions. Sequences of restriction sites are underlined.

| **Gene name** | **Accession number** | **Primers** | **Sequences** |
| --- | --- | --- | --- |
| *MrMYB1* | ADG21957 | Forward | CGCTCTAGAACTAGTGGATCCATGGAAGGCTCTTTAGGTGTA |
|  |  | Reverse | GATAAGCTTGATATCGAATTCTTATGGATCGAGAAAATCCCA |
| *MrMYB2* | MW835219 | Forward | CGCTCTAGAACTAGTGGATCCATGGAGCGCTCCTCCAGTGTGC |
|  |  | Reverse | GATAAGCTTGATATCGAATTCTTAGATCAGCGCTCCTTCTTCGGC |
| *MrMYB12* | KAB1200696.1 | Forward | CGCTCTAGAACTAGTGGATCCATGGGAAGGGCTCCGTGCT |
|  |  | Reverse | GATAAGCTTGATATCGAATTCTCAGGAAAGAAGCCAAGCAACCAT |
| *MrMYB111* | KAB1213881.1 | Forward | CGCTCTAGAACTAGTGGATCCATGGGAAGGGCACCATGTTGCG |
|  |  | Reverse | GATAAGCTTGATATCGAATTCTCACCTTTGCCAGCTTCCCATA |
| *MrMYB39* | KAB1218364.1 | Forward | CGCTCTAGAACTAGTGGATCCATGGCTTCCATGACAGATGG |
|  |  | Reverse | GATAAGCTTGATATCGAATTCGGCTTCTACAGTTTATAAGATATCAG |
| *MrMYB130* | KAB1209459.1 | Forward | CGCTCTAGAACTAGTGGATCCATGGGGAGAAGCCCTTGTTG |
|  |  | Reverse | GATAAGCTTGATATCGAATTCCACGGCCCGTATTGTATTAATTATT |
| *MrMYB58a/b* | KAB1219664.1/KAB1219671.1 | Forward | CGCTCTAGAACTAGTGGATCCAGGGAGATGGGAAGGGCT |
|  |  | Reverse | GATAAGCTTGATATCGAATTCTCAGATAAGTAATGATTCAGCGAAGG |
| *MrMYB40* | KAB1218366.1 | Forward | CGCTCTAGAACTAGTGGATCCATGGGAAGGAGTCCTTGTTGTTC |
|  |  | Reverse | GATAAGCTTGATATCGAATTCTCATAGCCAATCCAGTTCAGACTC |
| *MrMYB5* | KAB1223383.1 | Forward | CGCTCTAGAACTAGTGGATCCATGAAGAACCCATCATCAGGGGCAAGCAA |
|  |  | Reverse | GATAAGCTTGATATCGAATTCTCATGCTGGGTGATTGTTTTGCGCA |

**Table S3** Primers for promoter amplification and LUC vector constructions. Sequences of restriction sites are underlined.

| **Genes** | **Accession number** | **Primers** | **Sequences** |
| --- | --- | --- | --- |
| *MrDFR1* | KAB1203812.1 | Forward | CTATAGGGCGAATTGGGTACCATTGAGTTGGTCGGGATG |
|  |  | Reverse | CAGGAATTCGATATCAAGCTTGCTCCTGCTCCTCAC |
| *MrFLS1* | KAB1205367.1 | Forward | CTATAGGGCGAATTGGGTACCACGCTGGCTGGCTTCTTTACC |
|  |  | Reverse | CAGGAATTCGATATCAAGCTTAGGCCTGCACTCTCTCTACTTC |
| *MrLAR1* | KAB1221133.1 | Forward | CTATAGGGCGAATTGGGTACCAATTGTTTGGGCAGTTGAGC |
|  |  | Reverse | CAGGAATTCGATATCAAGCTTTTGTATCAAAAGAAAAGGGGC |
| *MrANR* | KAB1201247.1 | Forward | CTATAGGGCGAATTGGGTACCCCATAATTTGGGGAGTCATGTT |
|  |  | Reverse | CAGGAATTCGATATCAAGCTTGGACTTTGTTATTTATCGCTGC |

**Table S4** Primers used for quantitative real-time PCR analysis.

| **Gene** | **GenBank No.** | **Forward sequence (5**' **- 3**'**)** | **Reverse sequence (5**'**- 3**'**)** |
| --- | --- | --- | --- |
| *NtCHS* | EF421432 | AGAAAAGCCTTGTGGAAGCA | ACTTGGTCCAAAATTGCAGG |
| *NtCHI* | AB213651 | GAAATCCTCCGATCCAGTGA | CAACGTTGACAACATCAGGC |
| *NtF3H* | AB289450 | ACAGGGTGAAGTGGTCCAAG | CCTTGGTTAAGGCCTCCTTC |
| *NtF3'H* | AB289449 | TCCAAGAATACTGGCCCAAG | CTCACAACTCTCGGATGCAA |
| *NtF3'5'H* | XM_016597113 | GTGAAGATTGATCCAAGAGG | GTGCAGGACGAAGAATTTGTG |
| *NtFLS* | AB289451 | GAACTTGAAGGGAAAAGGGG | TCCCTGTAGGAGGGAGGATT |
| *NtDFR* | EF421431 | TCCCATCATGCGATCATCTA | ATGGCTTCTTTGTCACGTCC |
| *NtLAR* | AM827419 | TCAAGGTCCTTTACGCCATC | ACGAACCTGCTTCTCTTTGG |
| *NtANR1* | AM791704 | CATTTGACTTTCCCAAACGC | ATTGGGCTTTTGAGTTGTGC |
| *NtANR2* | DW003895 | TGTTCCCACTTGGGATGATA | TGCACCTATACTCTGTTAGTGGC |
| *NtANS* | AB289447 | TGGCGTTGAAGCTCATACTG | TTTCAAGGGTGTCCCCAATA |
| *NtUFGT* | FG627024 | GAGTGCATTGGATGCCTTTT | CCAGCTCCATTAGGTCCTTG |
| *NtEF1-α* | AF120093 | GCCCAACACTTCTTGATGCTC | GACACCAGTTTCCACACGAC |

**Table S5** The isoelectric point, molecular weight, chromosome location, and MYB-domain type of the members of *MrMYB* gene family. The principles of gene naming are as follows: 1. Gene names of the *MrMYB* locus have been reported; 2. *MrMYB* genes were named based on their homologous genes in *Arabidopsis thaliana*; 3. Without meeting the first and second principles, *MrMYB* genes were named according to the order of chromosome location.

| **Number** | **Gene name** | **Accession number** | **pl** | **MW(Da)** | **Chromosome** | **MYB-domain type** |
| --- | --- | --- | --- | --- | --- | --- |
| 1 | *MrMYB1* | KAB1211297.1 | 8.74 | 27981.84 | 6 | R2R3-MYB |
| 2 | *MrMYB2* | KAB1211298.1 | 8.16 | 28584.43 | 6 | R2R3-MYB |
| 3 | *MrMYB3* | KAB1210887.1 | 8.22 | 33782.2 | 6 | R2R3-MYB |
| 4 | *MrMYB4* | KAB1207546.1 | 8.88 | 27328.97 | 7 | R2R3-MYB |
| 5 | *MrMYB5* | KAB1223383.1 | 8.65 | 32443.35 | 2 | R2R3-MYB |
| 6 | *MrMYB6* | KAB1225415.1 | 6.12 | 33391.45 | 1 | 1R-MYB |
| 7 | *MrMYB7* | KAB1208929.1 | 8.99 | 34656.88 | 6 | R2R3-MYB |
| 8 | *MrMYB8* | KAB1225676.1 | 5.75 | 71361.03 | 1 | 1R-MYB |
| 9 | *MrMYB9* | KAB1225680.1 | 5.75 | 71361.03 | 1 | 1R-MYB |
| 10 | *MrMYB10* | KAB1225767.1 | 9.5 | 34868.19 | 1 | 1R-MYB |
| 11 | *MrMYB11* | KAB1225949.1 | 9.18 | 76042.1 | 1 | 1R-MYB |
| 12 | *MrMYB12* | KAB1200696.1 | 5.01 | 43832.93 | scaffold_272 | R2R3-MYB |
| 13 | *MrMYB13* | KAB1226354.1 | 4.43 | 8578.19 | 1 | 1R-MYB |
| 14 | *MrMYB14* | KAB1200935.1 | 5.41 | 31716.55 | scaffold_221 | R2R3-MYB |
| 15 | *MrMYB15* | KAB1219246.1 | 5.23 | 31337.94 | 3 | R2R3-MYB |
| 16 | *MrMYB16* | KAB1223011.1 | 5.97 | 42664.24 | 2 | R2R3-MYB |
| 17 | *MrMYB17a* | KAB1205236.1 | 5.86 | 35117.78 | 7 | R2R3-MYB |
| 18 | *MrMYB17b* | KAB1205240.1 | 5.86 | 35117.78 | 7 | R2R3-MYB |
| 19 | *MrMYB18* | KAB1226482.1 | 6.04 | 66200.4 | 1 | 1R-MYB |
| 20 | *MrMYB19* | KAB1227557.1 | 5.63 | 64652.1 | 1 | 1R-MYB |
| 21 | *MrMYB20a* | KAB1211323.1 | 4.96 | 34579.67 | 6 | R2R3-MYB |
| 22 | *MrMYB20b* | KAB1208087.1 | 4.83 | 33457.3 | 7 | R2R3-MYB |
| 23 | *MrMYB21* | KAB1209160.1 | 7.8 | 22774.54 | 6 | R2R3-MYB |
| 24 | *MrMYB22* | KAB1221586.1 | 8.35 | 106868.6 | 2 | R2R3-MYB |
| 25 | *MrMYB23* | KAB1222320.1 | 4.82 | 93135.99 | 2 | 1R-MYB |
| 26 | *MrMYB24* | KAB1223117.1 | 9.81 | 16096.53 | 2 | 1R-MYB |
| 27 | *MrMYB25* | KAB1224278.1 | 5.39 | 43322.23 | 2 | R2R3-MYB |
| 28 | *MrMYB26* | KAB1223816.1 | 6.51 | 56435.45 | 2 | 1R-MYB |
| 29 | *MrMYB27* | KAB1223602.1 | 5.6 | 30223.85 | 2 | R2R3-MYB |
| 30 | *MrMYB28* | KAB1223906.1 | 9.3 | 22390.32 | 2 | 1R-MYB |
| 31 | *MrMYB29* | KAB1223920.1 | 8.74 | 25748.34 | 2 | 1R-MYB |
| 32 | *MrMYB30* | KAB1224228.1 | 10.07 | 30758.31 | 2 | 1R-MYB |
| 33 | *MrMYB31* | KAB1224307.1 | 6.6 | 35821.02 | 2 | 1R-MYB |
| 34 | *MrMYB32* | KAB1224601.1 | 6.38 | 42096.54 | 2 | R2R3-MYB |
| 35 | *MrMYB33* | KAB1225022.1 | 5.22 | 60408.22 | 1 | R2R3-MYB |
| 36 | *MrMYB34* | KAB1217779.1 | 8.59 | 33002.14 | 3 | 1R-MYB |
| 37 | *MrMYB35* | KAB1211625.1 | 5.92 | 40624.54 | 6 | R2R3-MYB |
| 38 | *MrMYB36* | KAB1204208.1 | 6.12 | 41358.52 | 8 | R2R3-MYB |
| 39 | *MrMYB37* | KAB1217856.1 | 8.89 | 81319.14 | 3 | 1R-MYB |
| 40 | *MrMYB38a* | KAB1223531.1 | 7.58 | 36316.25 | 2 | R2R3-MYB |
| 41 | *MrMYB38b* | KAB1223532.1 | 7.58 | 36316.25 | 2 | R2R3-MYB |
| 42 | *MrMYB39* | KAB1218364.1 | 8.43 | 24288.23 | 3 | R2R3-MYB |
| 43 | *MrMYB40* | KAB1218366.1 | 8.11 | 32660.83 | 3 | R2R3-MYB |
| 44 | *MrMYB41* | KAB1218367.1 | 6.33 | 32207.23 | 3 | R2R3-MYB |
| 45 | *MrMYB42* | KAB1218374.1 | 8.3 | 74971.69 | 3 | 1R-MYB |
| 46 | *MrMYB43* | KAB1218395.1 | 9.5 | 30862.95 | 3 | 1R-MYB |
| 47 | *MrMYB44a* | KAB1217636.1 | 8.3 | 27812.22 | 3 | R2R3-MYB |
| 48 | *MrMYB44b* | KAB1217711.1 | 8.3 | 27812.22 | 3 | R2R3-MYB |
| 49 | *MrMYB45* | KAB1210711.1 | 6.86 | 33103.92 | 6 | R2R3-MYB |
| 50 | *MrMYB46a* | KAB1227655.1 | 6.31 | 33556.86 | 1 | R2R3-MYB |
| 51 | *MrMYB46b* | KAB1227663.1 | 6.31 | 33556.86 | 1 | R2R3-MYB |
| 52 | *MrMYB47* | KAB1218404.1 | 9.17 | 19986.93 | 3 | R2R3-MYB |
| 53 | *MrMYB48* | KAB1226039.1 | 6.25 | 29180.66 | 1 | R2R3-MYB |
| 54 | *MrMYB49* | KAB1218406.1 | 8.86 | 32575.04 | 3 | R2R3-MYB |
| 55 | *MrMYB50* | KAB1218407.1 | 6.72 | 39442.18 | 3 | R2R3-MYB |
| 56 | *MrMYB51* | KAB1218408.1 | 6.19 | 39183.82 | 3 | R2R3-MYB |
| 57 | *MrMYB52* | KAB1212181.1 | 8.71 | 28556.1 | 5 | R2R3-MYB |
| 58 | *MrMYB53* | KAB1218507.1 | 6.96 | 37310.9 | 3 | 1R-MYB |
| 59 | *MrMYB54* | KAB1218632.1 | 7.21 | 32020.17 | 3 | R2R3-MYB |
| 60 | *MrMYB55* | KAB1203457.1 | 7.17 | 50717.52 | 8 | R2R3-MYB |
| 61 | *MrMYB56* | KAB1219490.1 | 8.69 | 37637.02 | 3 | 1R-MYB |
| 62 | *MrMYB57* | KAB1219594.1 | 9.41 | 10621.16 | 3 | 1R-MYB |
| 63 | *MrMYB58a* | KAB1219664.1 | 9.04 | 32229.54 | 3 | R2R3-MYB |
| 64 | *MrMYB58b* | KAB1219671.1 | 9.04 | 32229.54 | 3 | R2R3-MYB |
| 65 | *MrMYB59* | KAB1226038.1 | 7.69 | 30150.8 | 1 | R2R3-MYB |
| 66 | *MrMYB60* | KAB1199932.1 | 6.27 | 35722.19 | scaffold_591 | R2R3-MYB |
| 67 | *MrMYB61* | KAB1199163.1 | 8.92 | 48830.67 | scaffold_924 | R2R3-MYB |
| 68 | *MrMYB62* | KAB1205541.1 | 7.17 | 34310.74 | 7 | R2R3-MYB |
| 69 | *MrMYB63* | KAB1216919.1 | 5.12 | 38851.04 | 4 | R2R3-MYB |
| 70 | *MrMYB64* | KAB1210660.1 | 7.04 | 54443.38 | 6 | R2R3-MYB |
| 71 | *MrMYB65* | KAB1227415.1 | 6.46 | 36645.18 | 1 | R2R3-MYB |
| 72 | *MrMYB66* | KAB1219785.1 | 6.65 | 32975.1 | 3 | R2R3-MYB |
| 73 | *MrMYB67* | KAB1220102.1 | 9.63 | 49825.26 | 3 | 1R-MYB |
| 74 | *MrMYB68* | KAB1220633.1 | 6.83 | 10317.48 | 3 | 1R-MYB |
| 75 | *MrMYB69* | KAB1210565.1 | 9.38 | 29551.34 | 6 | R2R3-MYB |
| 76 | *MrMYB70* | KAB1220312.1 | 8.6 | 33414.49 | 3 | R2R3-MYB |
| 77 | *MrMYB71* | KAB1219375.1 | 9.25 | 30135.79 | 3 | R2R3-MYB |
| 78 | *MrMYB72* | KAB1220639.1 | 9.05 | 11340.57 | 3 | 1R-MYB |
| 79 | *MrMYB73* | KAB1208437.1 | 9.5 | 29041.48 | 7 | R2R3-MYB |
| 80 | *MrMYB74* | KAB1210069.1 | 5.5 | 38919.87 | 6 | R2R3-MYB |
| 81 | *MrMYB75a* | KAB1215229.1 | 7.63 | 41361.57 | 4 | R2R3-MYB |
| 82 | *MrMYB75b* | KAB1215240.1 | 7.63 | 41361.57 | 4 | R2R3-MYB |
| 83 | *MrMYB75c* | KAB1215241.1 | 7.63 | 41361.57 | 4 | R2R3-MYB |
| 84 | *MrMYB76* | KAB1215275.1 | 6.52 | 39265.53 | 4 | Atypical MYB genes |
| 85 | *MrMYB77a* | KAB1220389.1 | 8.68 | 25587.47 | 3 | R2R3-MYB |
| 86 | *MrMYB77b* | KAB1220390.1 | 8.68 | 25587.47 | 3 | R2R3-MYB |
| 87 | *MrMYB78* | KAB1200199.1 | 6.17 | 32641.11 | scaffold_4 | R2R3-MYB |
| 88 | *MrMYB79* | KAB1215276.1 | 6.32 | 34690.3 | 4 | Atypical MYB genes |
| 89 | *MrMYB80* | KAB1215011.1 | 9.61 | 34829.77 | 4 | R2R3-MYB |
| 90 | *MrMYB81a* | KAB1215281.1 | 5.39 | 35035.31 | 4 | Atypical MYB genes |
| 91 | *MrMYB81b* | KAB1215298.1 | 5.39 | 35035.31 | 4 | Atypical MYB genes |
| 92 | *MrMYB82* | KAB1199375.1 | 7.05 | 28017.48 | scaffold_864 | R2R3-MYB |
| 93 | *MrMYB83* | KAB1224740.1 | 5.66 | 36037.28 | 1 | R2R3-MYB |
| 94 | *MrMYB84* | KAB1215321.1 | 4.41 | 8374.2 | 4 | 1R-MYB |
| 95 | *MrMYB85* | KAB1217064.1 | 5.07 | 30504.23 | 4 | R2R3-MYB |
| 96 | *MrMYB86* | KAB1215670.1 | 5.43 | 41410 | 4 | R2R3-MYB |
| 97 | *MrMYB87* | KAB1215514.1 | 9.23 | 31409.25 | 4 | R2R3-MYB |
| 98 | *MrMYB88* | KAB1216701.1 | 6.17 | 40885.33 | 4 | R2R3-MYB |
| 99 | *MrMYB89* | KAB1216738.1 | 6.66 | 35552.11 | 4 | R2R3-MYB |
| 100 | *MrMYB90* | KAB1216987.1 | 7.19 | 36188.96 | 4 | 1R-MYB |
| 101 | *MrMYB91* | KAB1224111.1 | 9.46 | 40943.77 | 2 | R2R3-MYB |
| 102 | *MrMYB92* | KAB1214917.1 | 9.7 | 31728.51 | 5 | 1R-MYB |
| 103 | *MrMYB93a* | KAB1225026.1 | 6.45 | 37341.14 | 1 | R2R3-MYB |
| 104 | *MrMYB93b* | KAB1210234.1 | 6.45 | 37341.14 | 6 | R2R3-MYB |
| 105 | *MrMYB94* | KAB1211607.1 | 6.12 | 36468.95 | 6 | R2R3-MYB |
| 106 | *MrMYB95* | KAB1212317.1 | 9.1 | 26689.35 | 5 | R2R3-MYB |
| 107 | *MrMYB96* | KAB1209451.1 | 6.09 | 36360.49 | 6 | R2R3-MYB |
| 108 | *MrMYB97* | KAB1212318.1 | 9.96 | 22878.28 | 5 | R2R3-MYB |
| 109 | *MrMYB98* | KAB1209153.1 | 5.91 | 28088.55 | 6 | R2R3-MYB |
| 110 | *MrMYB99* | KAB1212451.1 | 6.9 | 31479.8 | 5 | 1R-MYB |
| 111 | *MrMYB100* | KAB1212483.1 | 9.26 | 30871.52 | 5 | R2R3-MYB |
| 112 | *MrMYB101* | KAB1201975.1 | 6.21 | 56098.32 | 8 | R2R3-MYB |
| 113 | *MrMYB102* | KAB1221875.1 | 5.26 | 41045.72 | 2 | R2R3-MYB |
| 114 | *MrMYB103* | KAB1215385.1 | 5.38 | 35735.96 | 4 | R2R3-MYB |
| 115 | *MrMYB104* | KAB1212666.1 | 7.05 | 31884.67 | 5 | 1R-MYB |
| 116 | *MrMYB105* | KAB1212833.1 | 7.95 | 36981.94 | 5 | R2R3-MYB |
| 117 | *MrMYB106* | KAB1208247.1 | 7.1 | 41736.01 | 7 | R2R3-MYB |
| 118 | *MrMYB107* | KAB1212835.1 | 6.32 | 38066.77 | 5 | R2R3-MYB |
| 119 | *MrMYB108* | KAB1213803.1 | 6.17 | 36661.38 | 5 | R2R3-MYB |
| 120 | *MrMYB109* | KAB1217791.1 | 5.61 | 47981.73 | 3 | R2R3-MYB |
| 121 | *MrMYB110* | KAB1223147.1 | 8.32 | 45395.75 | 2 | R2R3-MYB |
| 122 | *MrMYB111* | KAB1213881.1 | 5.5 | 44656.93 | 5 | R2R3-MYB |
| 123 | *MrMYB112* | KAB1212902.1 | 9.26 | 30871.52 | 5 | R2R3-MYB |
| 124 | *MrMYB113a* | KAB1213085.1 | 10.08 | 15490.13 | 5 | R2R3-MYB |
| 125 | *MrMYB113b* | KAB1213330.1 | 10.08 | 15490.13 | 5 | R2R3-MYB |
| 126 | *MrMYB114* | KAB1214562.1 | 9 | 9793.78 | 5 | 1R-MYB |
| 127 | *MrMYB115* | KAB1214571.1 | 9.47 | 9629.02 | 5 | 1R-MYB |
| 128 | *MrMYB116* | KAB1212524.1 | 5.5 | 35252.12 | 5 | R2R3-MYB |
| 129 | *MrMYB117* | KAB1208741.1 | 6.25 | 51585.51 | 6 | 1R-MYB |
| 130 | *MrMYB118* | KAB1209240.1 | 8.57 | 25771.04 | 6 | R2R3-MYB |
| 131 | *MrMYB119* | KAB1228048.1 | 8.11 | 52187.91 | Chr4_plus | R2R3-MYB |
| 132 | *MrMYB120* | KAB1215186.1 | 7.32 | 55678.74 | 4 | R2R3-MYB |
| 133 | *MrMYB121* | KAB1211429.1 | 5.57 | 27875.08 | 6 | R2R3-MYB |
| 134 | *MrMYB122* | KAB1209267.1 | 6.94 | 31784.51 | 6 | R2R3-MYB |
| 135 | *MrMYB123* | KAB1209357.1 | 6.93 | 30276.04 | 6 | 1R-MYB |
| 136 | *MrMYB124* | KAB1208024.1 | 6.36 | 55784.56 | 7 | R2R3-MYB |
| 137 | *MrMYB125* | KAB1225219.1 | 5.44 | 37690.26 | 1 | R2R3-MYB |
| 138 | *MrMYB126* | KAB1209435.1 | 6.38 | 37863.77 | 6 | R2R3-MYB |
| 139 | *MrMYB127* | KAB1209436.1 | 8.69 | 36559.64 | 6 | R2R3-MYB |
| 140 | *MrMYB128* | KAB1209438.1 | 5.43 | 46409.04 | 6 | R2R3-MYB |
| 141 | *MrMYB129* | KAB1209441.1 | 5.95 | 35219.55 | 6 | R2R3-MYB |
| 142 | *MrMYB130* | KAB1209459.1 | 7.13 | 34778.09 | 6 | R2R3-MYB |
| 143 | *MrMYB131* | KAB1209460.1 | 6.8 | 35500.65 | 6 | R2R3-MYB |
| 144 | *MrMYB132* | KAB1209912.1 | 8.52 | 32922.94 | 6 | 1R-MYB |
| 145 | *MrMYB133* | KAB1210059.1 | 5.72 | 42729.52 | 6 | R2R3-MYB |
| 146 | *MrMYB134* | KAB1210060.1 | 7.81 | 29252.68 | 6 | R2R3-MYB |
| 147 | *MrMYB135* | KAB1210542.1 | 5.24 | 116886.34 | 6 | Atypical MYB genes |
| 148 | *MrMYB136* | KAB1210709.1 | 6.01 | 33772.84 | 6 | R2R3-MYB |
| 149 | *MrMYB137* | KAB1211168.1 | 9.26 | 43505.12 | 6 | R2R3-MYB |
| 150 | *MrMYB138* | KAB1211278.1 | 8.72 | 25613.07 | 6 | R2R3-MYB |
| 151 | *MrMYB139* | KAB1211294.1 | 7.7 | 28096.99 | 6 | R2R3-MYB |
| 152 | *MrMYB140* | KAB1211299.1 | 6.04 | 29025.7 | 6 | R2R3-MYB |
| 153 | *MrMYB141* | KAB1211364.1 | 9.18 | 85126.99 | 6 | 1R-MYB |
| 154 | *MrMYB142* | KAB1211430.1 | 6.2 | 29457.14 | 6 | R2R3-MYB |
| 155 | *MrMYB143* | KAB1204903.1 | 9.63 | 49825.26 | 7 | 1R-MYB |
| 156 | *MrMYB144* | KAB1205617.1 | 8.86 | 73101.11 | 7 | Atypical MYB genes |
| 157 | *MrMYB145* | KAB1207285.1 | 9.16 | 20282.06 | 7 | 1R-MYB |
| 158 | *MrMYB146* | KAB1207292.1 | 9.22 | 11577.01 | 7 | 1R-MYB |
| 159 | *MrMYB147* | KAB1207943.1 | 9.24 | 32840.52 | 7 | 1R-MYB |
| 160 | *MrMYB148* | KAB1202677.1 | 4.91 | 17653.31 | 8 | 1R-MYB |
| 161 | *MrMYB149* | KAB1203920.1 | 6.34 | 66154.11 | 8 | 1R-MYB |
| 162 | *MrMYB150* | KAB1204150.1 | 5.04 | 34586.13 | 8 | R2R3-MYB |
| 163 | *MrMYB151a* | KAB1228154.1 | 8.61 | 27481.33 | Chr2_plus | R2R3-MYB |
| 164 | *MrMYB151b* | KAB1228151.1 | 8.61 | 27481.33 | Chr2_plus | R2R3-MYB |
| 165 | *MrMYB152* | KAB1228145.1 | 5.34 | 21679.28 | chr4_2 | 1R-MYB |
| 166 | *MrMYB153* | KAB1201374.1 | 5.24 | 31990.71 | scaffold_121 | R2R3-MYB |
| 167 | *MrMYB154* | KAB1200773.1 | 7.22 | 25752.04 | scaffold_256 | R2R3-MYB |
| 168 | *MrMYB155* | KAB1200772.1 | 8.78 | 24304.38 | scaffold_256 | R2R3-MYB |
| 169 | *MrMYB156* | KAB1200770.1 | 10.19 | 18650.31 | scaffold_256 | R2R3-MYB |
| 170 | *MrMYB157a* | KAB1199039.1 | 10.16 | 9520.01 | scaffold_963 | R2R3-MYB |
| 171 | *MrMYB157b* | KAB1199038.1 | 10.16 | 9520.01 | scaffold_963 | R2R3-MYB |
| 172 | *MrMYB3R1* | KAB1204398.1 | 6.02 | 113581.71 | 8 | 3R-MYB |
| 173 | *MrMYB3R5* | KAB1224109.1 | 8.53 | 63760.53 | 2 | 3R-MYB |
| 174 | *MrMYB4R1* | KAB1212325.1 | 7.97 | 123695.73 | 5 | 4R-MYB |

**Table** **S6** The *Ks* value for tandemly and syntenically duplicated *MrMYB* genes.

| **Gene 1** | **Gene 2** | **Duplication type** | **Ks** |
| --- | --- | --- | --- |
| *MrMYB59* | *MrMYB48* | tandem | 0.048 |
| *MrMYB38a* | *MrMYB38b* | tandem | 0.000 |
| *MrMYB77a* | *MrMYB77b* | tandem | 0.000 |
| *MrMYB40* | *MrMYB41* | tandem | 0.526 |
| *MrMYB49* | *MrMYB50* | tandem | 0.105 |
| *MrMYB50* | *MrMYB51* | tandem | 0.052 |
| *MrMYB75b* | *MrMYB75c* | tandem | 0.000 |
| *MrMYB76* | *MrMYB79* | tandem | 0.001 |
| *MrMYB95* | *MrMYB97* | tandem | 2.046 |
| *MrMYB133* | *MrMYB134* | tandem | 2.512 |
| *MrMYB130* | *MrMYB131* | tandem | 0.764 |
| *MrMYB121* | *MrMYB142* | tandem | 0.960 |
| *MrMYB2* | *MrMYB140* | tandem | 0.684 |
| *MrMYB1* | *MrMYB2* | tandem | 0.654 |
| *MrMYB126* | *MrMYB127* | tandem | 0.133 |
| *MrMYB11* | *MrMYB37* | syntenic | 1.064 |
| *MrMYB25* | *MrMYB109* | syntenic | 1.723 |
| *MrMYB31* | *MrMYB34* | syntenic | 1.174 |
| *MrMYB44a* | *MrMYB44b* | syntenic | 0.000 |
| *MrMYB68* | *MrMYB115* | syntenic | 1.957 |
| *MrMYB72* | *MrMYB114* | syntenic | 1.304 |
| *MrMYB43* | *MrMYB123* | syntenic | 1.287 |
| *MrMYB39* | *MrMYB130* | syntenic | 2.293 |
| *MrMYB41* | *MrMYB129* | syntenic | 1.708 |
| *MrMYB56* | *MrMYB143* | syntenic | 1.687 |
| *MrMYB68* | *MrMYB145* | syntenic | 3.071 |
| *MrMYB72* | *MrMYB146* | syntenic | 1.076 |
| *MrMYB67* | *MrMYB143* | syntenic | 0.000 |
| *MrMYB120* | *MrMYB101* | syntenic | 2.809 |
| *MrMYB100* | *MrMYB112* | syntenic | 0.000 |
| *MrMYB20a* | *MrMYB20b* | syntenic | 0.000 |

**Table S7** Transcript levels of *MrMYB* genes in different tissues and during fruit development. Abbreviations: L, Leaf; Fl, Flower; Fr, Fruit.

| **Gene name** | **L1** | **L2** | **L3** | **Fl1** | **Fl2** | **Fl3** | **FrS1-1** | **FrS1-2** | **FrS1-3** | **FrS2-1** | **FrS2-2** | **FrS2-3** | **FrS3-1** | **FrS3-2** | **FrS3-3** | **FrS4-1** | **FrS4-2** | **FrS4-3** |
| --- | --- | --- | --- | --- | --- | --- | --- | --- | --- | --- | --- | --- | --- | --- | --- | --- | --- | --- |
| *MrMYB1* | 0.3 | 0.0 | 0.1 | 3.4 | 5.7 | 3.2 | 1.3 | 3.3 | 2.1 | 74.9 | 70.0 | 26.7 | 47.9 | 76.1 | 67.0 | 88.7 | 78.4 | 75.9 |
| *MrMYB2* | 0.1 | 0.0 | 0.0 | 1.3 | 0.7 | 0.5 | 0.0 | 0.0 | 4.5 | 1.8 | 3.4 | 1.9 | 9.2 | 14.1 | 15.6 | 8.8 | 1.8 | 13.2 |
| *MrMYB3* | 9.3 | 12.7 | 6.5 | 44.2 | 54.1 | 54.0 | 78.7 | 80.9 | 90.2 | 30.9 | 26.9 | 23.3 | 18.9 | 27.8 | 23.8 | 35.4 | 27.2 | 41.2 |
| *MrMYB4* | 10.4 | 12.8 | 12.9 | 41.5 | 47.2 | 30.5 | 3.0 | 3.8 | 3.5 | 24.0 | 18.6 | 17.4 | 13.1 | 19.4 | 18.5 | 20.1 | 16.9 | 12.6 |
| *MrMYB5* | 30.0 | 35.2 | 21.0 | 9.5 | 9.9 | 9.0 | 5.4 | 6.4 | 6.1 | 0.5 | 1.6 | 1.2 | 1.3 | 1.4 | 1.7 | 1.1 | 0.4 | 1.1 |
| *MrMYB6* | 31.4 | 29.7 | 34.9 | 15.2 | 14.1 | 33.8 | 10.3 | 9.2 | 13.5 | 4.5 | 6.1 | 5.3 | 9.3 | 7.6 | 10.1 | 14.7 | 4.0 | 23.5 |
| *MrMYB7* | 35.1 | 30.5 | 24.2 | 9.6 | 10.2 | 7.1 | 7.5 | 8.6 | 7.5 | 2.4 | 3.5 | 2.3 | 1.3 | 3.5 | 2.4 | 5.6 | 3.3 | 2.8 |
| *MrMYB8* | 5.1 | 6.5 | 5.3 | 7.3 | 7.0 | 8.6 | 4.9 | 4.6 | 4.3 | 2.7 | 3.5 | 2.8 | 3.9 | 4.2 | 2.8 | 2.2 | 2.3 | 2.5 |
| *MrMYB9* | 5.1 | 6.5 | 5.3 | 7.3 | 7.0 | 8.6 | 4.9 | 4.6 | 4.3 | 2.7 | 3.5 | 2.8 | 3.9 | 4.2 | 2.8 | 2.2 | 2.3 | 2.5 |
| *MrMYB10* | 23.0 | 27.2 | 23.4 | 18.0 | 15.0 | 20.1 | 12.0 | 16.0 | 14.5 | 14.4 | 12.5 | 16.2 | 15.5 | 14.4 | 19.8 | 12.1 | 13.0 | 11.9 |
| *MrMYB11* | 41.2 | 43.5 | 26.8 | 34.5 | 28.3 | 34.3 | 18.2 | 18.9 | 19.0 | 17.7 | 16.7 | 15.3 | 14.5 | 20.1 | 14.0 | 12.8 | 15.3 | 11.9 |
| *MrMYB12* | 1.0 | 0.5 | 0.2 | 21.3 | 22.2 | 20.8 | 0.4 | 0.6 | 1.1 | 0.1 | 0.3 | 0.0 | 0.2 | 0.4 | 0.2 | 0.1 | 0.0 | 0.3 |
| *MrMYB13* | 17.7 | 15.8 | 15.7 | 53.9 | 59.9 | 45.9 | 53.5 | 70.1 | 61.9 | 82.4 | 63.6 | 91.7 | 73.4 | 76.7 | 80.1 | 66.7 | 67.8 | 66.2 |
| *MrMYB14* | 1.0 | 0.3 | 1.2 | 3.5 | 1.8 | 4.2 | 2.1 | 1.0 | 0.1 | 0.1 | 0.0 | 0.0 | 0.4 | 0.9 | 0.9 | 22.8 | 2.2 | 21.3 |
| *MrMYB15* | 1.4 | 0.6 | 7.7 | 1.9 | 3.3 | 2.6 | 0.8 | 0.3 | 0.1 | 0.0 | 0.1 | 0.2 | 0.1 | 0.5 | 0.7 | 0.6 | 0.1 | 0.8 |
| *MrMYB16* | 20.6 | 28.4 | 13.4 | 64.5 | 75.4 | 56.8 | 3.0 | 2.8 | 1.4 | 0.0 | 0.0 | 0.0 | 0.1 | 0.1 | 0.0 | 0.0 | 0.1 | 0.0 |
| *MrMYB17a* | 1.5 | 3.7 | 1.2 | 44.1 | 44.6 | 39.2 | 7.9 | 9.6 | 10.9 | 1.6 | 1.3 | 1.1 | 0.5 | 0.6 | 1.0 | 0.3 | 0.1 | 0.0 |
| *MrMYB17b* | 1.5 | 3.7 | 1.2 | 44.1 | 44.6 | 39.2 | 7.9 | 9.6 | 10.9 | 1.6 | 1.3 | 1.1 | 0.5 | 0.6 | 1.0 | 0.3 | 0.1 | 0.0 |
| *MrMYB18* | 13.0 | 13.7 | 14.7 | 27.7 | 25.9 | 25.8 | 10.9 | 11.2 | 14.9 | 7.7 | 9.7 | 6.7 | 6.7 | 8.2 | 7.4 | 4.2 | 7.8 | 3.5 |
| *MrMYB19* | 0.0 | 0.0 | 0.0 | 0.0 | 0.0 | 0.0 | 0.0 | 0.0 | 0.0 | 0.0 | 0.0 | 0.0 | 0.0 | 0.0 | 0.0 | 0.0 | 0.0 | 0.0 |
| *MrMYB20a* | 341.4 | 227.4 | 237.9 | 16.8 | 19.6 | 13.9 | 3.9 | 2.7 | 2.3 | 9.2 | 8.1 | 11.2 | 10.4 | 13.7 | 10.8 | 21.9 | 18.1 | 22.4 |
| *MrMYB20b* | 0.0 | 0.0 | 0.0 | 0.0 | 0.0 | 0.0 | 0.0 | 0.0 | 0.0 | 0.0 | 0.0 | 0.0 | 0.0 | 0.0 | 0.0 | 0.0 | 0.0 | 0.0 |
| *MrMYB21* | 0.0 | 0.0 | 0.0 | 0.0 | 0.0 | 0.0 | 0.0 | 0.0 | 0.0 | 0.0 | 0.0 | 0.0 | 0.0 | 0.0 | 0.0 | 0.0 | 0.0 | 0.0 |
| *MrMYB22* | 9.2 | 8.2 | 7.2 | 13.3 | 10.6 | 14.7 | 8.5 | 8.4 | 9.3 | 4.8 | 5.7 | 2.3 | 2.1 | 3.5 | 3.5 | 2.2 | 4.1 | 2.9 |
| *MrMYB23* | 28.0 | 27.0 | 14.2 | 25.8 | 22.9 | 22.0 | 22.9 | 18.4 | 30.9 | 6.4 | 10.3 | 7.0 | 8.0 | 6.5 | 8.0 | 7.4 | 5.7 | 7.4 |
| *MrMYB24* | 82.3 | 66.6 | 32.3 | 227.9 | 229.6 | 182.8 | 56.9 | 69.5 | 82.4 | 45.0 | 38.2 | 30.4 | 32.4 | 22.5 | 25.2 | 11.7 | 30.8 | 19.0 |
| *MrMYB25* | 4.6 | 4.6 | 3.5 | 6.8 | 8.1 | 5.4 | 5.6 | 5.8 | 6.0 | 5.4 | 5.3 | 4.1 | 3.6 | 4.0 | 4.5 | 3.4 | 3.7 | 2.0 |
| *MrMYB26* | 52.6 | 45.2 | 41.8 | 68.1 | 57.9 | 66.9 | 33.7 | 39.3 | 39.3 | 26.0 | 30.2 | 24.2 | 21.9 | 25.6 | 20.7 | 17.9 | 20.1 | 16.4 |
| *MrMYB27* | 0.2 | 0.5 | 0.4 | 1.2 | 0.5 | 0.6 | 1.6 | 0.8 | 0.5 | 0.0 | 0.0 | 0.0 | 0.0 | 0.0 | 0.0 | 0.1 | 0.3 | 0.1 |
| *MrMYB28* | 0.0 | 0.0 | 0.0 | 0.0 | 0.0 | 0.0 | 0.0 | 0.0 | 0.0 | 0.0 | 0.0 | 0.0 | 0.0 | 0.0 | 0.0 | 0.0 | 0.0 | 0.0 |
| *MrMYB29* | 31.9 | 26.8 | 24.1 | 20.2 | 16.4 | 27.0 | 11.7 | 16.0 | 16.1 | 4.5 | 4.7 | 3.5 | 2.5 | 4.4 | 3.4 | 1.5 | 2.0 | 2.0 |
| *MrMYB30* | 0.0 | 0.0 | 0.0 | 5.1 | 3.6 | 3.1 | 0.0 | 0.1 | 0.0 | 0.0 | 0.0 | 0.0 | 0.0 | 0.0 | 0.0 | 0.0 | 0.0 | 0.0 |
| *MrMYB31* | 6.9 | 9.7 | 9.1 | 12.7 | 11.8 | 11.8 | 9.2 | 9.5 | 9.1 | 17.5 | 20.9 | 39.6 | 33.5 | 25.8 | 34.6 | 17.8 | 21.7 | 21.2 |
| *MrMYB32* | 0.0 | 0.0 | 0.0 | 0.0 | 0.0 | 1.6 | 0.0 | 0.0 | 0.0 | 0.0 | 0.0 | 0.0 | 0.0 | 0.0 | 0.0 | 0.0 | 0.0 | 0.0 |
| *MrMYB33* | 15.5 | 10.8 | 9.2 | 25.3 | 20.8 | 22.1 | 13.2 | 12.2 | 13.7 | 10.9 | 13.7 | 7.4 | 7.8 | 10.5 | 7.2 | 6.6 | 9.0 | 6.0 |
| *MrMYB34* | 3.0 | 4.6 | 2.8 | 1.6 | 2.2 | 0.7 | 3.5 | 6.4 | 7.4 | 0.7 | 2.6 | 3.2 | 1.1 | 2.1 | 2.6 | 1.0 | 1.5 | 1.8 |
| *MrMYB35* | 0.0 | 0.0 | 0.0 | 0.0 | 0.0 | 0.0 | 0.0 | 0.0 | 0.0 | 0.0 | 0.0 | 0.0 | 0.0 | 0.0 | 0.0 | 0.0 | 0.0 | 0.0 |
| *MrMYB36* | 0.0 | 0.0 | 0.0 | 1.3 | 0.0 | 0.0 | 0.0 | 3.9 | 0.0 | 0.0 | 0.0 | 0.0 | 0.0 | 0.0 | 0.0 | 0.0 | 0.0 | 0.0 |
| *MrMYB37* | 42.1 | 41.6 | 41.5 | 30.7 | 23.4 | 48.7 | 24.9 | 31.5 | 41.9 | 23.5 | 19.2 | 18.4 | 21.0 | 25.0 | 16.7 | 36.1 | 28.4 | 37.1 |
| *MrMYB38a* | 6.7 | 6.2 | 8.1 | 21.9 | 25.3 | 25.6 | 9.6 | 9.5 | 9.5 | 2.1 | 4.5 | 6.8 | 7.2 | 5.5 | 5.8 | 2.6 | 2.9 | 2.2 |
| *MrMYB38b* | 6.7 | 6.2 | 8.1 | 21.9 | 25.3 | 25.6 | 9.6 | 9.5 | 9.5 | 2.1 | 4.5 | 6.8 | 7.2 | 5.5 | 5.8 | 2.6 | 2.9 | 2.2 |
| *MrMYB39* | 4.8 | 10.3 | 4.8 | 7.8 | 9.9 | 10.5 | 0.4 | 0.3 | 0.2 | 0.0 | 0.1 | 0.3 | 0.7 | 0.0 | 0.1 | 0.1 | 0.0 | 0.0 |
| *MrMYB40* | 28.5 | 34.2 | 42.4 | 38.3 | 41.2 | 34.4 | 1.4 | 0.8 | 1.0 | 0.6 | 0.9 | 0.1 | 1.0 | 2.0 | 1.3 | 0.5 | 1.3 | 0.2 |
| *MrMYB41* | 0.0 | 0.0 | 0.0 | 0.0 | 0.0 | 0.0 | 0.0 | 0.0 | 0.0 | 0.0 | 0.0 | 0.0 | 0.0 | 0.0 | 0.0 | 0.0 | 0.0 | 0.0 |
| *MrMYB42* | 44.0 | 41.1 | 30.5 | 51.1 | 42.4 | 44.2 | 39.9 | 39.4 | 45.4 | 34.1 | 45.6 | 28.6 | 28.2 | 33.1 | 24.2 | 24.4 | 32.3 | 22.7 |
| *MrMYB43* | 5.5 | 4.9 | 3.3 | 16.3 | 13.8 | 14.5 | 4.5 | 3.3 | 5.9 | 4.4 | 3.8 | 3.1 | 3.3 | 3.1 | 2.3 | 3.3 | 3.1 | 1.8 |
| *MrMYB44a* | 0.0 | 0.0 | 0.0 | 0.1 | 0.0 | 0.2 | 0.5 | 0.3 | 0.0 | 0.0 | 0.0 | 0.2 | 0.4 | 0.0 | 0.9 | 0.4 | 0.0 | 0.0 |
| *MrMYB44b* | 0.0 | 0.0 | 0.0 | 0.1 | 0.0 | 0.2 | 0.5 | 0.3 | 0.0 | 0.0 | 0.0 | 0.2 | 0.4 | 0.0 | 0.9 | 0.4 | 0.0 | 0.0 |
| *MrMYB45* | 0.0 | 3.2 | 0.0 | 0.0 | 0.0 | 0.0 | 0.0 | 0.0 | 0.0 | 5.8 | 0.0 | 0.0 | 0.0 | 0.0 | 0.0 | 0.0 | 0.0 | 0.0 |
| *MrMYB46a* | 1.1 | 1.1 | 0.1 | 0.6 | 0.5 | 0.5 | 0.1 | 0.0 | 0.1 | 0.3 | 0.0 | 0.0 | 0.0 | 0.0 | 0.0 | 0.0 | 0.0 | 0.0 |
| *MrMYB46b* | 1.1 | 1.1 | 0.1 | 0.6 | 0.5 | 0.5 | 0.1 | 0.0 | 0.1 | 0.3 | 0.0 | 0.0 | 0.0 | 0.0 | 0.0 | 0.0 | 0.0 | 0.0 |
| *MrMYB47* | 0.0 | 0.0 | 0.0 | 0.0 | 0.0 | 0.0 | 0.0 | 0.0 | 0.0 | 0.0 | 0.0 | 0.0 | 0.0 | 0.0 | 0.0 | 0.0 | 0.0 | 0.0 |
| *MrMYB48* | 1.0 | 1.6 | 1.5 | 9.5 | 6.1 | 11.4 | 19.2 | 13.0 | 15.2 | 1.5 | 1.3 | 1.4 | 1.4 | 1.2 | 1.3 | 0.8 | 0.5 | 1.0 |
| *MrMYB49* | 0.0 | 0.0 | 0.0 | 0.0 | 0.0 | 0.0 | 0.0 | 0.0 | 0.0 | 0.0 | 0.0 | 0.0 | 0.0 | 0.0 | 0.0 | 0.0 | 0.0 | 0.0 |
| *MrMYB50* | 0.0 | 0.0 | 0.0 | 0.0 | 0.0 | 0.0 | 0.0 | 0.0 | 0.0 | 0.0 | 0.0 | 0.0 | 0.0 | 0.0 | 0.0 | 0.0 | 0.0 | 0.0 |
| *MrMYB51* | 5.1 | 0.6 | 0.1 | 8.7 | 5.1 | 9.0 | 364.9 | 399.7 | 339.9 | 1624.9 | 1796.0 | 1559.6 | 1390.7 | 1400.3 | 1384.4 | 1129.3 | 1405.1 | 1054.3 |
| *MrMYB52* | 6.0 | 8.5 | 7.7 | 5.2 | 4.3 | 5.3 | 16.3 | 23.5 | 13.1 | 5.1 | 5.0 | 6.6 | 4.5 | 7.5 | 6.9 | 4.5 | 3.5 | 3.2 |
| *MrMYB53* | 161.0 | 118.5 | 297.0 | 28.2 | 24.8 | 75.5 | 9.9 | 9.6 | 9.6 | 7.4 | 6.7 | 8.6 | 10.8 | 7.8 | 10.6 | 24.6 | 12.3 | 26.7 |
| *MrMYB54* | 0.0 | 0.0 | 0.0 | 3.7 | 2.2 | 0.4 | 0.0 | 0.0 | 0.0 | 0.0 | 0.0 | 0.0 | 0.0 | 0.0 | 0.0 | 0.0 | 0.0 | 0.0 |
| *MrMYB55* | 10.4 | 12.6 | 7.8 | 16.8 | 17.6 | 13.7 | 9.7 | 10.7 | 12.8 | 9.1 | 14.5 | 18.1 | 14.0 | 15.7 | 16.6 | 9.3 | 8.9 | 9.0 |
| *MrMYB56* | 53.5 | 48.1 | 57.6 | 18.0 | 15.1 | 25.3 | 13.7 | 15.3 | 16.1 | 20.7 | 20.4 | 17.4 | 21.5 | 23.9 | 19.7 | 26.1 | 25.5 | 32.8 |
| *MrMYB57* | 8.6 | 13.5 | 12.3 | 16.1 | 18.3 | 18.2 | 4.5 | 5.4 | 4.4 | 3.3 | 4.1 | 1.5 | 2.5 | 4.1 | 4.1 | 2.0 | 3.9 | 2.3 |
| *MrMYB58a* | 5.1 | 7.1 | 15.3 | 61.6 | 57.2 | 48.7 | 3.0 | 0.6 | 0.3 | 0.1 | 0.2 | 0.3 | 0.1 | 0.1 | 0.1 | 0.4 | 1.2 | 0.1 |
| *MrMYB58b* | 5.1 | 7.1 | 15.3 | 61.6 | 57.2 | 48.7 | 3.0 | 0.6 | 0.3 | 0.1 | 0.2 | 0.3 | 0.1 | 0.1 | 0.1 | 0.4 | 1.2 | 0.1 |
| *MrMYB59* | 1.5 | 1.1 | 0.3 | 6.7 | 5.6 | 5.9 | 1.9 | 3.2 | 3.0 | 0.8 | 1.0 | 0.3 | 0.1 | 0.1 | 0.3 | 0.0 | 0.6 | 0.0 |
| *MrMYB60* | 26.0 | 26.1 | 11.0 | 65.0 | 73.4 | 67.3 | 33.6 | 29.1 | 24.0 | 8.8 | 14.8 | 11.6 | 12.6 | 12.6 | 10.5 | 7.1 | 5.6 | 4.9 |
| *MrMYB61* | 1.3 | 1.9 | 1.4 | 1.5 | 5.3 | 0.7 | 2.8 | 2.3 | 2.0 | 1.1 | 1.8 | 2.3 | 1.3 | 1.9 | 1.6 | 0.2 | 1.9 | 0.3 |
| *MrMYB62* | 2.7 | 1.7 | 5.8 | 12.1 | 19.1 | 16.4 | 17.6 | 8.3 | 7.8 | 40.2 | 34.0 | 39.1 | 41.7 | 44.3 | 51.1 | 71.9 | 47.3 | 51.0 |
| *MrMYB63* | 5.4 | 7.2 | 4.4 | 3.2 | 4.1 | 4.8 | 2.1 | 1.3 | 1.2 | 4.3 | 3.7 | 1.5 | 3.1 | 5.7 | 2.1 | 5.9 | 3.9 | 3.6 |
| *MrMYB64* | 0.0 | 0.0 | 0.0 | 0.0 | 0.0 | 0.0 | 0.0 | 0.0 | 0.0 | 0.0 | 0.0 | 0.0 | 0.0 | 0.0 | 0.0 | 0.0 | 0.0 | 0.0 |
| *MrMYB65* | 4.1 | 3.6 | 2.4 | 7.6 | 5.5 | 5.9 | 3.1 | 2.6 | 3.9 | 2.4 | 2.3 | 0.9 | 1.5 | 1.3 | 1.3 | 1.4 | 1.9 | 1.7 |
| *MrMYB66* | 0.6 | 1.0 | 1.3 | 2.9 | 4.9 | 3.9 | 0.8 | 0.8 | 0.7 | 0.0 | 0.7 | 0.0 | 0.0 | 0.3 | 0.4 | 0.4 | 0.1 | 0.0 |
| *MrMYB67* | 47.2 | 34.8 | 65.5 | 49.1 | 65.1 | 40.2 | 25.7 | 31.4 | 23.9 | 70.4 | 42.7 | 58.3 | 54.1 | 53.0 | 60.3 | 59.4 | 56.2 | 55.5 |
| *MrMYB68* | 0.0 | 0.0 | 0.0 | 0.0 | 0.0 | 0.0 | 0.0 | 0.0 | 0.0 | 0.0 | 0.0 | 0.0 | 0.0 | 0.0 | 0.0 | 1.9 | 0.0 | 2.9 |
| *MrMYB69* | 0.0 | 0.0 | 0.0 | 0.0 | 0.1 | 0.0 | 1.3 | 1.8 | 1.7 | 3.6 | 5.9 | 3.6 | 2.2 | 1.9 | 3.2 | 0.1 | 0.7 | 0.0 |
| *MrMYB70* | 66.4 | 70.1 | 111.1 | 47.7 | 65.6 | 53.6 | 22.6 | 22.4 | 15.2 | 26.7 | 17.0 | 24.7 | 29.7 | 25.9 | 32.1 | 61.4 | 44.6 | 53.9 |
| *MrMYB71* | 0.2 | 0.4 | 0.0 | 2.8 | 2.0 | 4.2 | 5.9 | 5.7 | 10.2 | 0.1 | 0.0 | 0.2 | 0.1 | 0.3 | 0.2 | 0.1 | 0.0 | 0.0 |
| *MrMYB72* | 3.0 | 4.7 | 3.1 | 1.1 | 0.9 | 1.0 | 4.9 | 5.4 | 4.0 | 9.0 | 7.2 | 10.9 | 11.0 | 12.7 | 12.9 | 19.4 | 24.0 | 21.1 |
| *MrMYB73* | 6.3 | 7.0 | 10.9 | 16.7 | 23.2 | 18.2 | 9.6 | 12.6 | 12.1 | 2.8 | 0.9 | 0.9 | 2.1 | 2.4 | 2.2 | 16.0 | 3.6 | 11.4 |
| *MrMYB74* | 0.0 | 0.0 | 0.0 | 0.8 | 0.0 | 0.0 | 0.0 | 0.0 | 0.0 | 0.0 | 0.0 | 0.0 | 0.0 | 0.0 | 0.0 | 0.0 | 0.0 | 0.0 |
| *MrMYB75a* | 0.2 | 0.0 | 1.1 | 2.6 | 3.9 | 2.6 | 0.7 | 1.4 | 1.0 | 0.2 | 1.6 | 0.1 | 0.3 | 0.1 | 0.1 | 1.9 | 0.6 | 1.3 |
| *MrMYB75b* | 0.2 | 0.0 | 1.1 | 2.6 | 3.9 | 2.6 | 0.7 | 1.4 | 1.0 | 0.2 | 1.6 | 0.1 | 0.3 | 0.1 | 0.1 | 1.9 | 0.6 | 1.3 |
| *MrMYB75c* | 0.2 | 0.0 | 1.1 | 2.6 | 3.9 | 2.6 | 0.7 | 1.4 | 1.0 | 0.2 | 1.6 | 0.1 | 0.3 | 0.1 | 0.1 | 1.9 | 0.6 | 1.3 |
| *MrMYB76* | 0.0 | 0.0 | 0.0 | 0.0 | 0.0 | 0.0 | 0.0 | 0.0 | 0.0 | 0.0 | 0.0 | 0.0 | 0.0 | 0.0 | 0.0 | 0.0 | 0.0 | 0.0 |
| *MrMYB77a* | 1.6 | 5.9 | 7.7 | 3.2 | 5.0 | 4.9 | 1.4 | 1.1 | 1.1 | 0.3 | 0.3 | 1.3 | 3.4 | 1.6 | 2.8 | 8.4 | 3.6 | 9.3 |
| *MrMYB77b* | 1.6 | 5.9 | 7.7 | 3.2 | 5.0 | 4.9 | 1.4 | 1.1 | 1.1 | 0.3 | 0.3 | 1.3 | 3.4 | 1.6 | 2.8 | 8.4 | 3.6 | 9.3 |
| *MrMYB78* | 0.3 | 0.2 | 0.2 | 5.1 | 7.0 | 10.4 | 1.9 | 0.2 | 0.3 | 0.0 | 0.5 | 0.1 | 0.8 | 0.8 | 0.5 | 5.2 | 2.5 | 6.6 |
| *MrMYB79* | 0.0 | 0.0 | 0.0 | 0.0 | 0.0 | 0.0 | 0.0 | 0.0 | 0.0 | 0.0 | 0.0 | 0.0 | 0.0 | 0.0 | 0.0 | 0.0 | 0.0 | 0.0 |
| *MrMYB80* | 0.0 | 0.0 | 0.0 | 0.0 | 0.0 | 0.0 | 0.0 | 0.0 | 0.0 | 0.0 | 7.9 | 0.0 | 0.0 | 0.0 | 0.0 | 0.0 | 0.0 | 0.0 |
| *MrMYB81a* | 0.5 | 1.4 | 0.5 | 2.2 | 1.8 | 4.1 | 1.5 | 1.1 | 0.5 | 0.2 | 0.2 | 1.2 | 0.5 | 0.2 | 0.1 | 0.0 | 0.0 | 0.1 |
| *MrMYB81b* | 0.5 | 1.4 | 0.5 | 2.2 | 1.8 | 4.1 | 1.5 | 1.1 | 0.5 | 0.2 | 0.2 | 1.2 | 0.5 | 0.2 | 0.1 | 0.0 | 0.0 | 0.1 |
| *MrMYB82* | 0.0 | 0.0 | 0.1 | 10.7 | 9.5 | 7.7 | 0.7 | 0.0 | 0.0 | 0.0 | 0.1 | 0.0 | 0.0 | 0.0 | 0.0 | 0.0 | 0.3 | 0.0 |
| *MrMYB83* | 0.7 | 2.1 | 0.6 | 0.2 | 2.8 | 0.6 | 0.0 | 0.0 | 0.0 | 0.0 | 0.0 | 0.0 | 0.0 | 0.0 | 0.0 | 0.0 | 0.0 | 0.0 |
| *MrMYB84* | 0.1 | 0.2 | 0.2 | 10.1 | 11.7 | 14.5 | 3.1 | 2.2 | 0.8 | 3.3 | 4.0 | 3.1 | 2.2 | 5.3 | 5.4 | 1.9 | 1.4 | 1.3 |
| *MrMYB85* | 6.5 | 16.0 | 3.3 | 1.1 | 1.5 | 0.5 | 0.3 | 0.2 | 0.4 | 0.2 | 0.3 | 0.2 | 0.4 | 0.1 | 0.3 | 0.1 | 0.1 | 0.1 |
| *MrMYB86* | 0.9 | 3.5 | 2.2 | 2.2 | 1.4 | 5.5 | 0.0 | 0.0 | 0.0 | 0.0 | 0.1 | 0.0 | 0.1 | 0.0 | 0.2 | 0.1 | 0.1 | 0.1 |
| *MrMYB87* | 0.3 | 0.1 | 0.1 | 3.8 | 5.7 | 3.2 | 0.0 | 0.0 | 0.0 | 0.7 | 0.5 | 2.8 | 0.8 | 1.6 | 1.2 | 0.7 | 0.4 | 0.5 |
| *MrMYB88* | 0.0 | 0.0 | 0.0 | 0.0 | 8.6 | 0.0 | 0.0 | 0.0 | 0.0 | 0.0 | 0.0 | 0.0 | 0.0 | 0.0 | 0.0 | 4.9 | 0.0 | 0.0 |
| *MrMYB89* | 0.1 | 0.0 | 0.0 | 33.2 | 43.3 | 29.2 | 0.0 | 0.0 | 0.0 | 0.0 | 0.0 | 0.0 | 0.0 | 0.0 | 0.0 | 0.0 | 0.0 | 0.0 |
| *MrMYB90* | 311.3 | 348.3 | 247.1 | 164.5 | 176.4 | 232.9 | 520.7 | 482.8 | 359.0 | 182.8 | 114.6 | 134.3 | 177.8 | 127.9 | 193.4 | 114.3 | 145.7 | 106.8 |
| *MrMYB91* | 29.7 | 38.7 | 25.8 | 105.4 | 99.8 | 88.8 | 57.1 | 66.7 | 71.4 | 45.0 | 43.8 | 56.0 | 56.3 | 44.2 | 59.3 | 24.5 | 35.2 | 24.1 |
| *MrMYB92* | 20.2 | 19.9 | 17.6 | 28.9 | 24.3 | 23.8 | 11.9 | 15.8 | 14.3 | 15.5 | 18.4 | 16.5 | 15.2 | 15.1 | 12.7 | 16.0 | 17.9 | 18.7 |
| *MrMYB93a* | 0.6 | 0.5 | 0.2 | 4.0 | 3.5 | 2.6 | 0.4 | 0.7 | 0.2 | 0.0 | 0.0 | 0.0 | 0.0 | 0.0 | 0.0 | 0.1 | 0.0 | 0.0 |
| *MrMYB93b* | 0.6 | 0.5 | 0.2 | 4.0 | 3.5 | 2.6 | 0.4 | 0.7 | 0.2 | 0.0 | 0.0 | 0.0 | 0.0 | 0.0 | 0.0 | 0.1 | 0.0 | 0.0 |
| *MrMYB94* | 2.6 | 4.9 | 2.8 | 17.1 | 23.9 | 23.3 | 8.8 | 7.8 | 6.6 | 23.7 | 19.4 | 19.4 | 17.5 | 19.8 | 17.8 | 17.9 | 17.1 | 11.4 |
| *MrMYB95* | 7.9 | 11.6 | 7.7 | 14.6 | 17.2 | 11.3 | 0.7 | 0.8 | 0.8 | 0.6 | 0.2 | 0.6 | 1.5 | 1.1 | 1.6 | 2.0 | 0.8 | 1.4 |
| *MrMYB96* | 18.9 | 21.0 | 13.7 | 9.7 | 11.2 | 10.8 | 2.3 | 3.4 | 3.0 | 1.0 | 1.1 | 1.8 | 2.3 | 4.3 | 2.0 | 1.6 | 1.0 | 2.5 |
| *MrMYB97* | 4.3 | 4.3 | 3.7 | 13.4 | 8.1 | 8.1 | 0.3 | 0.7 | 0.5 | 0.2 | 0.1 | 0.1 | 0.0 | 0.0 | 0.2 | 0.0 | 0.0 | 0.1 |
| *MrMYB98* | 0.0 | 0.0 | 0.0 | 0.0 | 0.0 | 0.0 | 0.0 | 0.0 | 0.0 | 0.0 | 0.0 | 0.0 | 0.0 | 0.0 | 0.0 | 0.0 | 0.0 | 0.0 |
| *MrMYB99* | 22.7 | 22.3 | 22.1 | 19.4 | 17.2 | 17.8 | 7.3 | 6.2 | 4.7 | 5.1 | 3.9 | 4.0 | 4.0 | 4.7 | 4.3 | 6.9 | 6.1 | 8.2 |
| *MrMYB100* | 0.1 | 0.2 | 0.0 | 20.1 | 21.8 | 17.8 | 0.0 | 0.0 | 0.1 | 0.0 | 0.0 | 0.0 | 0.0 | 0.0 | 0.0 | 0.2 | 0.0 | 0.2 |
| *MrMYB101* | 0.0 | 0.0 | 0.0 | 0.0 | 0.0 | 0.0 | 0.0 | 0.1 | 0.1 | 0.2 | 0.5 | 1.0 | 0.6 | 0.4 | 0.1 | 0.0 | 0.0 | 0.0 |
| *MrMYB102* | 28.2 | 23.9 | 18.5 | 23.5 | 13.2 | 33.1 | 0.5 | 0.8 | 1.9 | 0.7 | 0.5 | 1.0 | 0.4 | 0.7 | 0.6 | 0.8 | 0.3 | 1.0 |
| *MrMYB103* | 3.2 | 11.8 | 1.4 | 1.0 | 0.9 | 0.1 | 0.1 | 0.6 | 0.1 | 0.1 | 0.0 | 0.5 | 0.1 | 0.0 | 0.0 | 0.0 | 0.0 | 0.0 |
| *MrMYB104* | 25.6 | 24.3 | 9.0 | 24.1 | 31.8 | 25.9 | 14.9 | 15.5 | 16.5 | 13.6 | 15.8 | 10.3 | 7.7 | 6.1 | 5.2 | 13.4 | 16.4 | 13.4 |
| *MrMYB105* | 0.2 | 0.0 | 0.5 | 0.3 | 0.8 | 0.0 | 0.1 | 0.1 | 0.3 | 0.0 | 0.0 | 0.0 | 0.0 | 0.0 | 0.0 | 0.0 | 0.0 | 0.0 |
| *MrMYB106* | 0.1 | 0.2 | 0.0 | 20.1 | 21.8 | 17.8 | 0.0 | 0.0 | 0.1 | 0.0 | 0.0 | 0.0 | 0.0 | 0.0 | 0.0 | 0.2 | 0.0 | 0.2 |
| *MrMYB107* | 0.0 | 0.0 | 0.0 | 0.0 | 0.0 | 0.0 | 0.0 | 0.0 | 0.0 | 0.0 | 0.0 | 0.0 | 0.0 | 0.0 | 0.0 | 0.0 | 0.0 | 0.0 |
| *MrMYB108* | 20.2 | 12.6 | 26.0 | 27.7 | 17.4 | 49.3 | 2.0 | 1.7 | 2.7 | 0.4 | 0.2 | 0.4 | 1.4 | 1.9 | 1.0 | 11.4 | 2.9 | 6.8 |
| *MrMYB109* | 12.4 | 14.6 | 10.0 | 9.1 | 10.8 | 10.7 | 11.9 | 12.5 | 12.5 | 6.9 | 6.9 | 6.5 | 7.6 | 8.9 | 7.6 | 5.8 | 5.0 | 4.8 |
| *MrMYB110* | 1.4 | 0.4 | 2.7 | 2.6 | 2.1 | 1.5 | 0.2 | 0.0 | 0.0 | 2.7 | 1.9 | 1.8 | 2.5 | 4.3 | 2.6 | 2.7 | 2.5 | 1.7 |
| *MrMYB111* | 0.1 | 0.0 | 0.0 | 36.7 | 45.3 | 34.3 | 0.0 | 0.0 | 0.0 | 0.0 | 0.0 | 0.0 | 0.0 | 0.1 | 0.0 | 0.0 | 0.0 | 0.0 |
| *MrMYB112* | 0.1 | 0.2 | 0.0 | 20.1 | 21.8 | 17.8 | 0.0 | 0.0 | 0.1 | 0.0 | 0.0 | 0.0 | 0.0 | 0.0 | 0.0 | 0.2 | 0.0 | 0.2 |
| *MrMYB113a* | 0.0 | 0.0 | 0.0 | 0.0 | 0.0 | 0.0 | 0.0 | 0.0 | 0.0 | 0.0 | 0.0 | 0.0 | 0.0 | 0.0 | 0.0 | 0.0 | 0.0 | 0.0 |
| *MrMYB113b* | 0.0 | 0.0 | 0.0 | 0.0 | 0.0 | 0.0 | 0.0 | 0.0 | 0.0 | 0.0 | 0.0 | 0.0 | 0.0 | 0.0 | 0.0 | 0.0 | 0.0 | 0.0 |
| *MrMYB114* | 0.0 | 0.2 | 0.1 | 0.9 | 2.1 | 0.1 | 8.1 | 3.5 | 9.2 | 0.0 | 0.0 | 0.1 | 0.0 | 0.0 | 0.0 | 0.0 | 0.0 | 0.0 |
| *MrMYB115* | 0.8 | 0.4 | 0.7 | 5.3 | 9.5 | 5.8 | 0.3 | 0.0 | 0.0 | 0.0 | 0.3 | 0.0 | 0.4 | 0.3 | 0.6 | 0.0 | 1.1 | 0.3 |
| *MrMYB116* | 4.5 | 4.2 | 11.6 | 55.0 | 51.9 | 68.9 | 50.4 | 52.0 | 57.3 | 27.6 | 23.6 | 17.1 | 24.7 | 35.8 | 31.7 | 39.3 | 22.6 | 38.0 |
| *MrMYB117* | 7.7 | 7.1 | 4.8 | 8.5 | 9.6 | 9.3 | 3.2 | 4.1 | 4.6 | 4.6 | 5.5 | 4.8 | 5.2 | 4.7 | 3.3 | 3.5 | 17.1 | 4.5 |
| *MrMYB118* | 0.0 | 0.0 | 0.0 | 0.0 | 0.0 | 0.0 | 0.0 | 0.0 | 0.0 | 0.0 | 0.0 | 0.0 | 0.0 | 0.0 | 0.0 | 0.0 | 0.0 | 0.0 |
| *MrMYB119* | 0.0 | 0.0 | 0.0 | 0.0 | 0.0 | 0.0 | 0.0 | 0.0 | 0.0 | 0.0 | 0.0 | 0.0 | 0.0 | 0.0 | 0.0 | 0.0 | 0.0 | 0.0 |
| *MrMYB120* | 2.7 | 1.9 | 2.6 | 2.0 | 2.1 | 2.5 | 1.1 | 1.0 | 0.1 | 2.6 | 3.4 | 2.0 | 3.5 | 4.9 | 2.8 | 3.7 | 1.9 | 3.7 |
| *MrMYB121* | 0.7 | 0.5 | 1.7 | 0.0 | 0.0 | 0.3 | 0.0 | 0.0 | 0.0 | 0.0 | 0.0 | 0.0 | 0.0 | 0.0 | 0.0 | 0.0 | 0.0 | 0.0 |
| *MrMYB122* | 9.9 | 5.2 | 11.7 | 19.9 | 22.5 | 24.7 | 3.4 | 0.8 | 1.3 | 0.1 | 0.0 | 0.1 | 0.3 | 0.2 | 0.3 | 0.0 | 0.1 | 0.3 |
| *MrMYB123* | 12.6 | 14.8 | 13.2 | 15.8 | 14.5 | 16.9 | 7.7 | 11.2 | 8.6 | 9.3 | 8.9 | 7.4 | 15.9 | 11.6 | 6.1 | 5.4 | 7.4 | 4.3 |
| *MrMYB124* | 0.6 | 0.6 | 0.2 | 3.1 | 1.9 | 1.6 | 3.7 | 2.4 | 6.7 | 6.2 | 6.0 | 10.7 | 1.7 | 3.7 | 2.0 | 2.8 | 4.6 | 2.5 |
| *MrMYB125* | 0.0 | 0.0 | 0.0 | 0.0 | 0.0 | 0.0 | 0.0 | 0.0 | 0.0 | 0.0 | 0.0 | 0.0 | 0.0 | 0.0 | 0.0 | 0.0 | 0.0 | 0.0 |
| *MrMYB126* | 0.0 | 0.0 | 0.0 | 0.0 | 0.0 | 0.0 | 0.0 | 0.0 | 0.0 | 0.0 | 0.0 | 0.0 | 0.0 | 0.0 | 0.0 | 0.0 | 0.0 | 0.0 |
| *MrMYB127* | 0.0 | 0.0 | 0.0 | 0.0 | 0.0 | 0.0 | 0.0 | 0.0 | 0.0 | 0.0 | 0.0 | 0.0 | 0.0 | 0.0 | 0.0 | 0.0 | 0.0 | 0.0 |
| *MrMYB128* | 0.0 | 0.0 | 0.0 | 0.0 | 0.0 | 0.0 | 0.0 | 0.0 | 0.0 | 0.0 | 0.0 | 0.0 | 0.0 | 0.0 | 0.0 | 0.0 | 0.0 | 0.0 |
| *MrMYB129* | 0.0 | 0.0 | 0.0 | 0.0 | 0.0 | 0.0 | 0.0 | 0.0 | 0.0 | 0.0 | 0.0 | 0.0 | 0.0 | 0.0 | 0.0 | 0.0 | 0.0 | 0.0 |
| *MrMYB130* | 103.7 | 61.4 | 61.7 | 98.7 | 85.6 | 65.6 | 0.6 | 0.4 | 0.3 | 0.0 | 0.1 | 0.0 | 0.1 | 1.1 | 0.0 | 0.1 | 0.0 | 0.0 |
| *MrMYB131* | 0.0 | 0.0 | 0.0 | 0.0 | 0.0 | 0.0 | 0.0 | 0.0 | 0.0 | 0.0 | 0.0 | 0.0 | 0.0 | 0.0 | 0.0 | 0.0 | 0.0 | 0.0 |
| *MrMYB132* | 50.5 | 56.1 | 44.5 | 101.5 | 106.1 | 108.9 | 70.2 | 70.9 | 71.0 | 64.7 | 50.6 | 66.3 | 58.3 | 60.4 | 73.7 | 67.2 | 58.3 | 62.6 |
| *MrMYB133* | 3.9 | 3.0 | 15.4 | 7.7 | 5.9 | 8.6 | 0.6 | 0.7 | 0.3 | 0.0 | 0.0 | 0.0 | 0.0 | 0.0 | 0.0 | 0.0 | 0.0 | 0.1 |
| *MrMYB134* | 0.0 | 0.0 | 0.0 | 0.0 | 0.0 | 0.0 | 0.0 | 0.0 | 0.0 | 0.0 | 0.0 | 0.0 | 0.0 | 0.0 | 0.0 | 0.0 | 0.0 | 0.0 |
| *MrMYB135* | 28.9 | 27.3 | 23.1 | 26.6 | 22.8 | 19.9 | 29.9 | 25.2 | 27.5 | 16.5 | 22.0 | 16.9 | 21.7 | 22.0 | 15.4 | 16.6 | 19.9 | 14.8 |
| *MrMYB136* | 0.1 | 0.1 | 0.0 | 5.2 | 5.1 | 5.3 | 7.1 | 12.2 | 8.1 | 0.5 | 2.5 | 3.0 | 2.2 | 1.3 | 0.7 | 2.9 | 0.6 | 2.2 |
| *MrMYB137* | 0.5 | 0.3 | 0.8 | 25.7 | 25.8 | 25.3 | 25.3 | 31.5 | 27.3 | 12.3 | 14.2 | 26.1 | 21.8 | 23.2 | 26.1 | 16.6 | 18.7 | 20.2 |
| *MrMYB138* | 44.9 | 48.3 | 25.4 | 47.6 | 51.0 | 47.3 | 52.2 | 74.3 | 66.7 | 11.3 | 14.3 | 10.1 | 12.1 | 15.5 | 13.2 | 10.9 | 14.7 | 11.7 |
| *MrMYB139* | 0.0 | 0.0 | 0.0 | 0.0 | 0.0 | 0.0 | 0.0 | 0.0 | 0.0 | 0.0 | 0.0 | 0.0 | 0.0 | 0.0 | 0.0 | 0.0 | 0.0 | 0.0 |
| *MrMYB140* | 0.0 | 0.0 | 0.0 | 0.0 | 0.0 | 0.0 | 0.0 | 0.0 | 0.0 | 0.0 | 0.0 | 0.0 | 0.0 | 0.0 | 0.0 | 0.0 | 0.0 | 0.0 |
| *MrMYB141* | 23.8 | 28.2 | 25.0 | 10.8 | 8.7 | 12.7 | 19.8 | 30.2 | 47.0 | 9.1 | 18.9 | 11.4 | 14.4 | 19.6 | 15.7 | 9.2 | 8.1 | 6.6 |
| *MrMYB142* | 0.0 | 0.1 | 0.6 | 0.0 | 0.0 | 0.0 | 0.1 | 0.0 | 0.2 | 0.9 | 0.1 | 0.0 | 0.8 | 1.7 | 1.0 | 4.7 | 8.2 | 4.2 |
| *MrMYB143* | 47.2 | 34.8 | 65.5 | 49.1 | 65.1 | 40.2 | 25.7 | 31.4 | 23.9 | 70.4 | 42.7 | 58.3 | 54.1 | 53.0 | 60.3 | 59.4 | 56.2 | 55.5 |
| *MrMYB144* | 11.6 | 10.7 | 8.9 | 11.7 | 10.6 | 6.8 | 4.1 | 4.4 | 4.9 | 3.3 | 6.9 | 6.5 | 4.3 | 6.2 | 4.9 | 4.3 | 4.9 | 4.5 |
| *MrMYB145* | 0.2 | 0.0 | 0.0 | 2.5 | 0.0 | 0.3 | 0.0 | 0.0 | 0.0 | 0.0 | 0.0 | 0.1 | 0.0 | 0.0 | 0.0 | 0.0 | 0.0 | 0.0 |
| *MrMYB146* | 3.2 | 2.8 | 3.2 | 12.9 | 14.9 | 10.6 | 14.8 | 19.5 | 22.3 | 5.5 | 12.7 | 19.6 | 13.1 | 6.8 | 18.5 | 3.5 | 4.8 | 2.4 |
| *MrMYB147* | 117.6 | 115.2 | 98.7 | 83.9 | 63.0 | 100.7 | 38.3 | 44.3 | 45.8 | 79.7 | 83.8 | 110.7 | 124.8 | 123.3 | 154.6 | 100.0 | 94.9 | 101.1 |
| *MrMYB148* | 0.0 | 0.0 | 0.0 | 0.0 | 0.0 | 0.0 | 0.0 | 0.0 | 0.0 | 0.0 | 0.0 | 0.0 | 0.0 | 0.0 | 0.0 | 0.0 | 0.0 | 0.0 |
| *MrMYB149* | 17.9 | 21.6 | 20.4 | 41.1 | 31.7 | 34.6 | 15.6 | 13.3 | 15.8 | 12.3 | 13.5 | 8.5 | 8.7 | 12.8 | 9.9 | 7.4 | 9.3 | 8.7 |
| *MrMYB150* | 0.0 | 0.0 | 0.0 | 0.0 | 0.0 | 0.0 | 0.0 | 0.0 | 0.0 | 0.0 | 0.0 | 0.0 | 0.0 | 0.0 | 0.0 | 0.0 | 0.0 | 0.0 |
| *MrMYB151a* | 0.0 | 0.0 | 0.0 | 3.1 | 2.9 | 3.4 | 0.5 | 2.4 | 1.1 | 1.1 | 3.0 | 0.4 | 0.2 | 0.2 | 1.5 | 0.0 | 0.1 | 0.0 |
| *MrMYB151b* | 0.0 | 0.0 | 0.0 | 3.1 | 2.9 | 3.4 | 0.5 | 2.4 | 1.1 | 1.1 | 3.0 | 0.4 | 0.2 | 0.2 | 1.5 | 0.0 | 0.1 | 0.0 |
| *MrMYB152* | 0.0 | 0.0 | 0.0 | 0.0 | 0.0 | 0.0 | 0.0 | 0.0 | 0.0 | 0.0 | 0.0 | 0.0 | 0.0 | 0.0 | 0.0 | 0.0 | 0.0 | 0.0 |
| *MrMYB153* | 0.0 | 0.0 | 0.0 | 1.2 | 0.8 | 0.8 | 0.0 | 0.0 | 0.0 | 0.0 | 0.0 | 0.0 | 0.0 | 0.0 | 0.0 | 0.0 | 0.0 | 0.0 |
| *MrMYB154* | 0.8 | 0.3 | 0.0 | 28.2 | 29.8 | 26.6 | 0.8 | 1.2 | 2.1 | 0.6 | 0.1 | 0.0 | 0.0 | 0.4 | 0.2 | 0.6 | 0.2 | 0.1 |
| *MrMYB155* | 0.0 | 0.0 | 0.0 | 12.9 | 13.5 | 12.2 | 0.0 | 0.0 | 0.0 | 0.0 | 0.0 | 0.0 | 0.0 | 0.0 | 0.0 | 0.0 | 0.0 | 0.0 |
| *MrMYB156* | 0.0 | 0.0 | 0.0 | 0.0 | 0.0 | 0.0 | 0.0 | 0.0 | 0.0 | 0.0 | 0.0 | 0.0 | 0.0 | 0.0 | 0.0 | 0.0 | 0.0 | 0.0 |
| *MrMYB157a* | 0.0 | 0.0 | 0.0 | 0.0 | 0.0 | 0.0 | 0.0 | 0.0 | 0.0 | 0.0 | 0.0 | 0.0 | 0.0 | 0.0 | 0.0 | 0.0 | 0.0 | 0.0 |
| *MrMYB157b* | 0.0 | 0.0 | 0.0 | 0.0 | 0.0 | 0.0 | 0.0 | 0.0 | 0.0 | 0.0 | 0.0 | 0.0 | 0.0 | 0.0 | 0.0 | 0.0 | 0.0 | 0.0 |
| *MrMYB3R1* | 4.1 | 4.0 | 2.9 | 16.4 | 12.2 | 11.8 | 3.1 | 2.6 | 3.7 | 3.5 | 4.6 | 3.5 | 2.7 | 4.4 | 2.8 | 1.5 | 2.9 | 2.6 |
| *MrMYB3R5* | 0.0 | 0.0 | 0.0 | 0.0 | 0.0 | 0.0 | 0.0 | 0.0 | 0.0 | 0.0 | 0.0 | 0.0 | 0.0 | 0.0 | 0.0 | 0.0 | 0.0 | 0.0 |
| *MrMYB4R1* | 3.6 | 2.8 | 3.3 | 5.1 | 3.3 | 3.9 | 2.4 | 2.4 | 2.0 | 2.9 | 2.7 | 1.8 | 1.5 | 1.7 | 1.9 | 1.6 | 2.1 | 1.9 |

**Table S8** Correlation between flavonoid-related *MrMYB* genes with flavonols, anthocyanins or proanthocyanidins contents. Expression patterns of the *MrMYB* genes and flavonoid profiles in different tissues and during fruit development are shown in Figure 4 and 5.

| **MYB clade** | **Gene name** | **Correlation (r, *P*)** | | |
| --- | --- | --- | --- | --- |
|  |  | **vs flavonols** | **vs anthocyanins** | **vs Proanthocyanidins** |
| Flavonols | *MrMYB12* (F1) | 0.82 (0.000) | - | - |
|  | *MrMYB111* (F2) | 0.81 (0.000) | - | - |
| Anthocyanins | *MrMYB1* (A1) | - | 0.83 (0.000) | - |
|  | *MrMYB2* (A2) | - | 0.73 (0.000) | - |
|  | *MrMYB139* (A3) | - | N/A | - |
|  | *MrMYB140* (A4) | - | N/A | - |
| Proanthocyanidins | *MrMYB40* (P1) | - | - | 0.98 (0.000) |
|  | *MrMYB39* (P2) | - | - | 0.96 (0.000) |
|  | *MrMYB130* (P3) | - | - | 0.95 (0.000) |
|  | *MrMYB58a/b* (P4/5) | - | - | 0.86 (0.000) |
|  | *MrMYB87* (P6) | - | - | 0.59 (0.003) |
|  | *MrMYB151a/b* (P7/8) | - | - | 0.41 (0.087) |
|  | *MrMYB41* (P9) | - | - | N/A |
|  | *MrMYB126* (P10) | - | - | N/A |
|  | *MrMYB127* (P11) | - | - | N/A |
|  | *MrMYB128* (P12) | - | - | N/A |
|  | *MrMYB129* (P13) | - | - | N/A |
|  | *MrMYB131* (P14) | - | - | N/A |
| Flavonoids | *MrMYB5* (Fd1) | 0.59 (0.008) | -0.64 (0.005) | 0.66 (0.003) |

**Table S9** Numbers of plant MYBs in the four different classes.

| **Species** | **1R-MYB** | **R2R3-MYB** | **3R-MYB** | **4R-MYB** | **Refs** |
| --- | --- | --- | --- | --- | --- |
| *Arabidopsis thaliana* | 64 | 126 | 5 | 1 | Chen et al., 2006 |
| *Citrus sinensis* | 90 | 85 | 1 | 1 | Hou et al., 2014 |
| *Glycine max* | 127 | 244 | 6 | 2 | Du et al., 2012; Du et al., 2013 |
| *Vitis vinifera* | 47 | 118 | 5 | n.d. | Wilkins et al., 2009; Du et al., 2013 |
| *Oryza sativa* | 70 | 109 | 4 | n.d. | Chen et al., 2006 |
| *Pyrus bretschneideri* | 22 | 105 | 2 | n.d. | Cao et al., 2016 |
| *Morella rubra* | 43 | 122 | 2 | 1 |  |

Abbreviations: n.d., not determined.

**Figure S1** The sequence logos of the R2 (A) and R3 (B) MYB repeats. These logos were based on the multiple sequence alignment of 122 R2R3-MYBs in Chinese bayberry. The core bits indicate the information content for each position in the sequence. The asterisks indicate the typical conserved Trp residues in the MYB domain. The triangle indicates the residue in the R3 repeat with position identical to the first conserved Trp residue in the R2 repeat.**
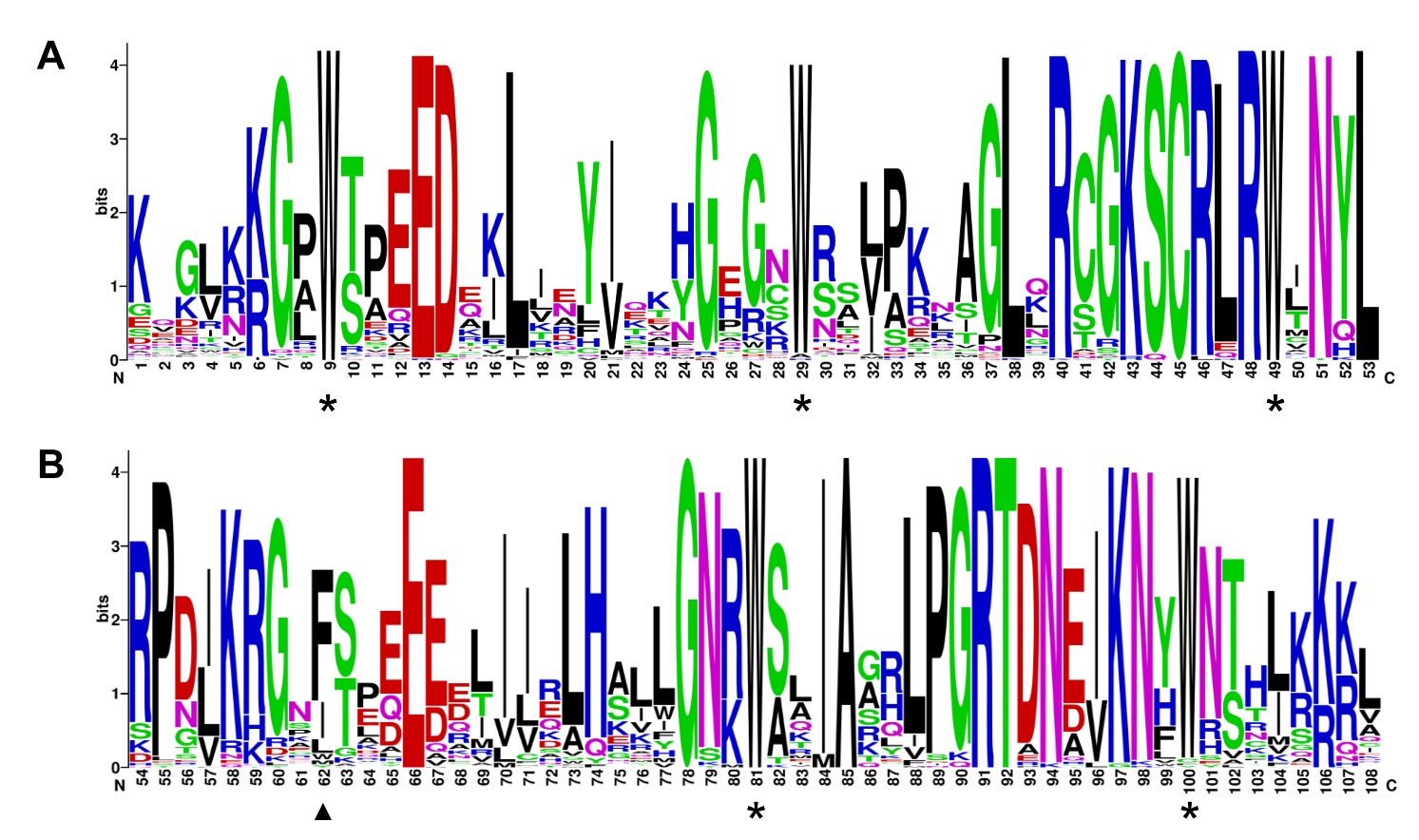
**

**Figure S2** Phylogenetic relationships (A), conserved motifs (B), and gene structure analysis (C) in Chinese bayberry 1R-, 3R- and 4R-MYBs. A Neighbor-joining phylogenetic tree was constructed by aligning the full-length amino acid sequences of 1R-, 3R- and 4R-MYBs. The seven subgroups are shown in different colors. The blue boxes and black lines in the exon-intron structure diagram represent exons and introns, respectively. The ten conserved motifs are exhibited with different colors and their specific sequence information is provided in Figure S3.

**
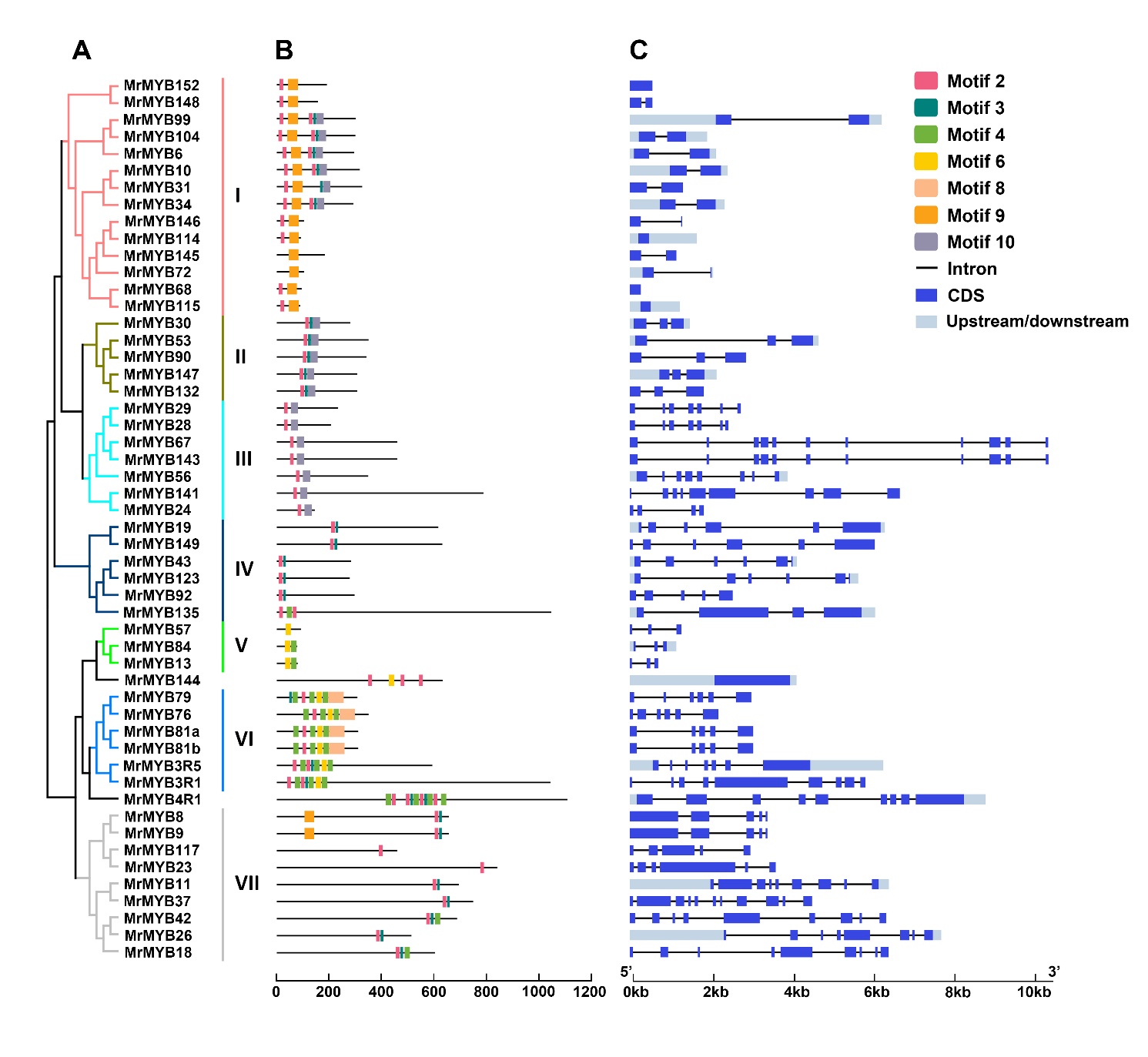
**

**Figure S3** All MEME motif sequence logos in MrMYBs.


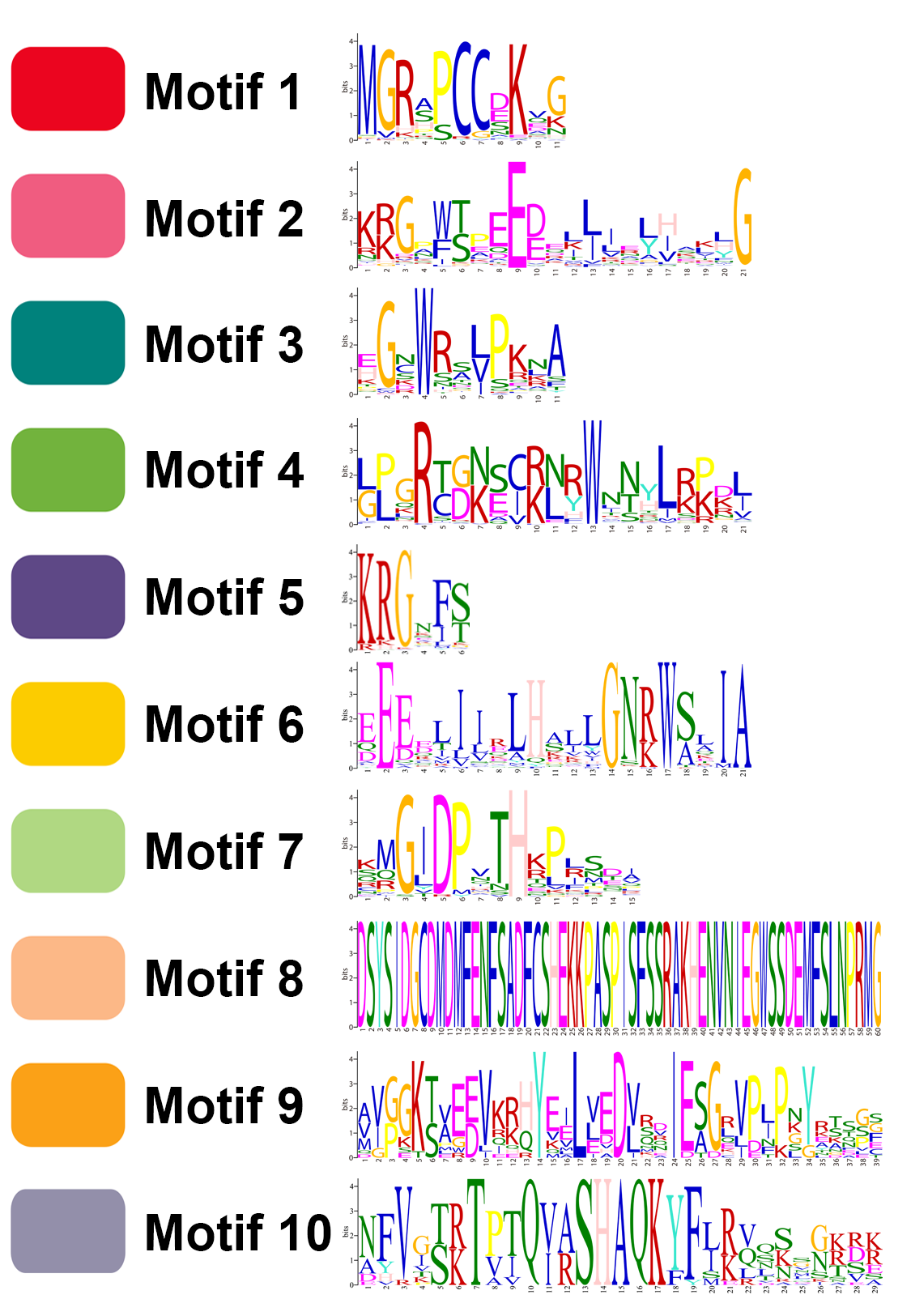


**Figure S4** Schematic representations for the interchromosomal relationships of *MrMYB* genes. (A) Grey lines in the background mean collinear blocks within Chinese bayberry, red lines indicate syntenic *MYB* gene pairs. (B) duplicated gene pairs of *MrMYB* genes are sorted according to their assigned MYB classes.


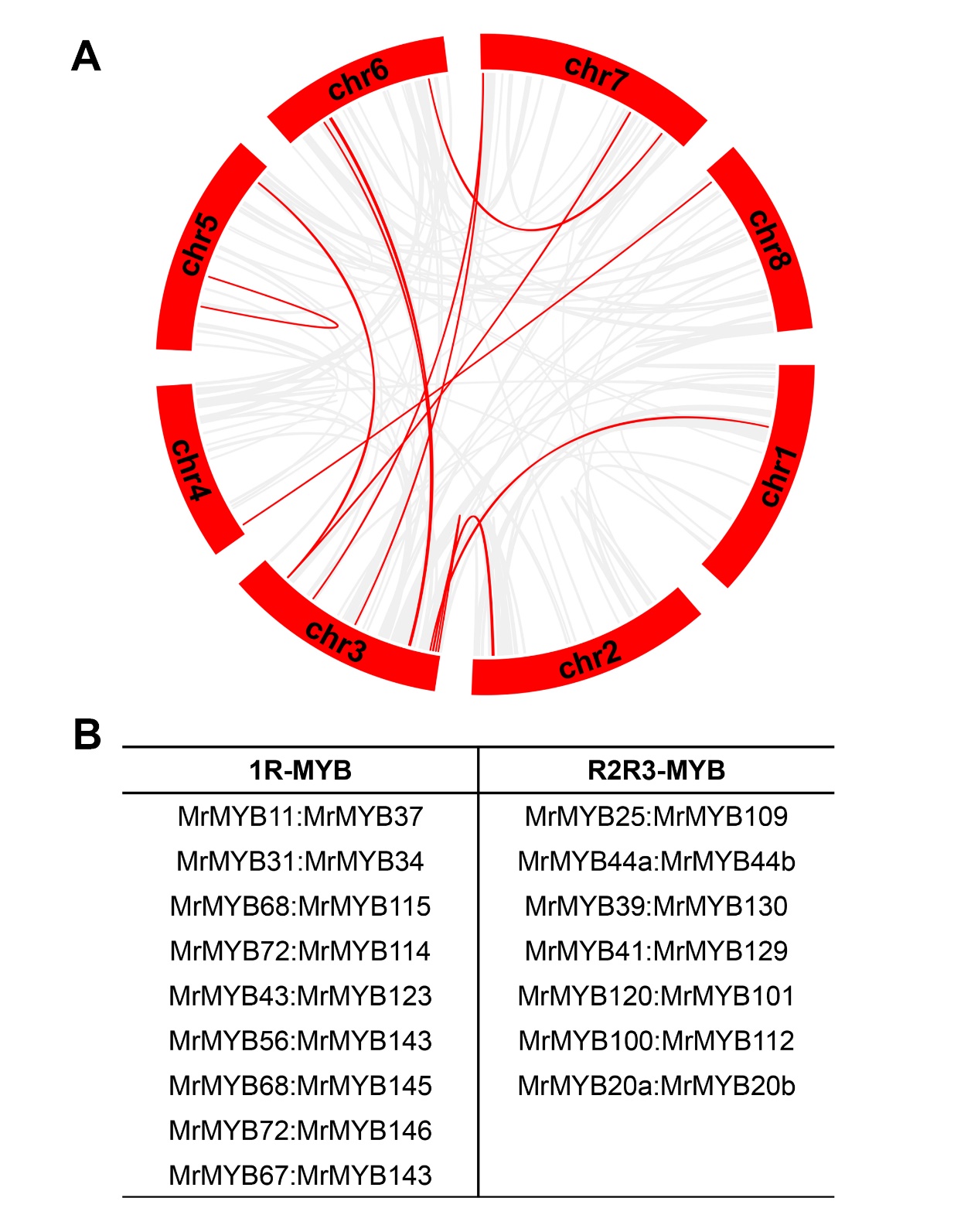


**Figure S5** Syntenic analysis of *MYB* genes between Chinese bayberry and *Juglans regia* (A), *Pyrus bretschneideri* (B), *Prunus persica* (C), *Medicago truncatula* (D), or *Arabidopsis thaliana* (E). Grey lines in the background mean collinear blocks between Chinese bayberry and other plant genomes, and red lines indicate syntenic *MYB* gene pairs.


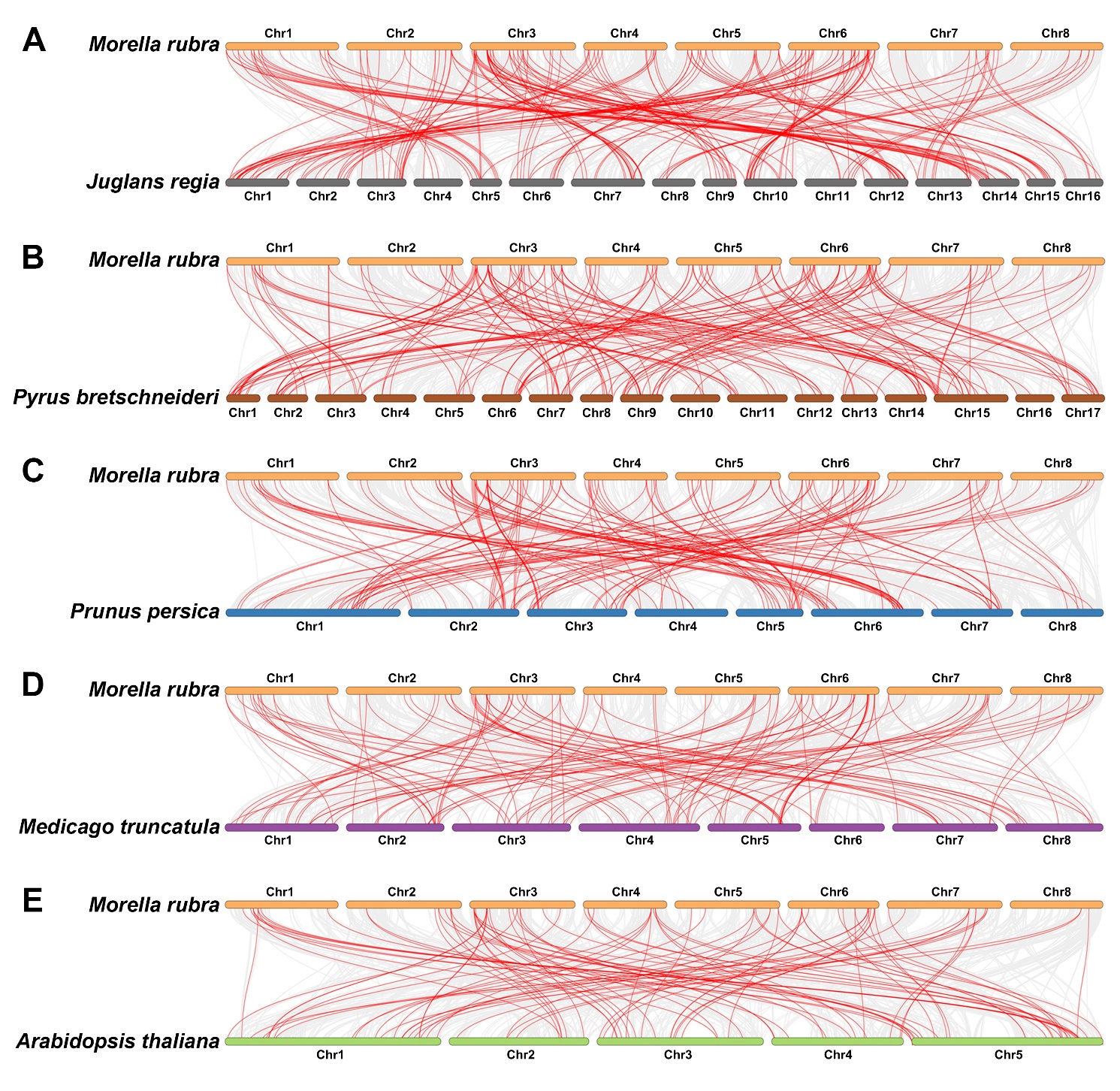


**Figure S6** Phylogenetic analysis of all MrMYBs and functional flavonoid-related MYB proteins from other plants. The clades are shown in different colors.


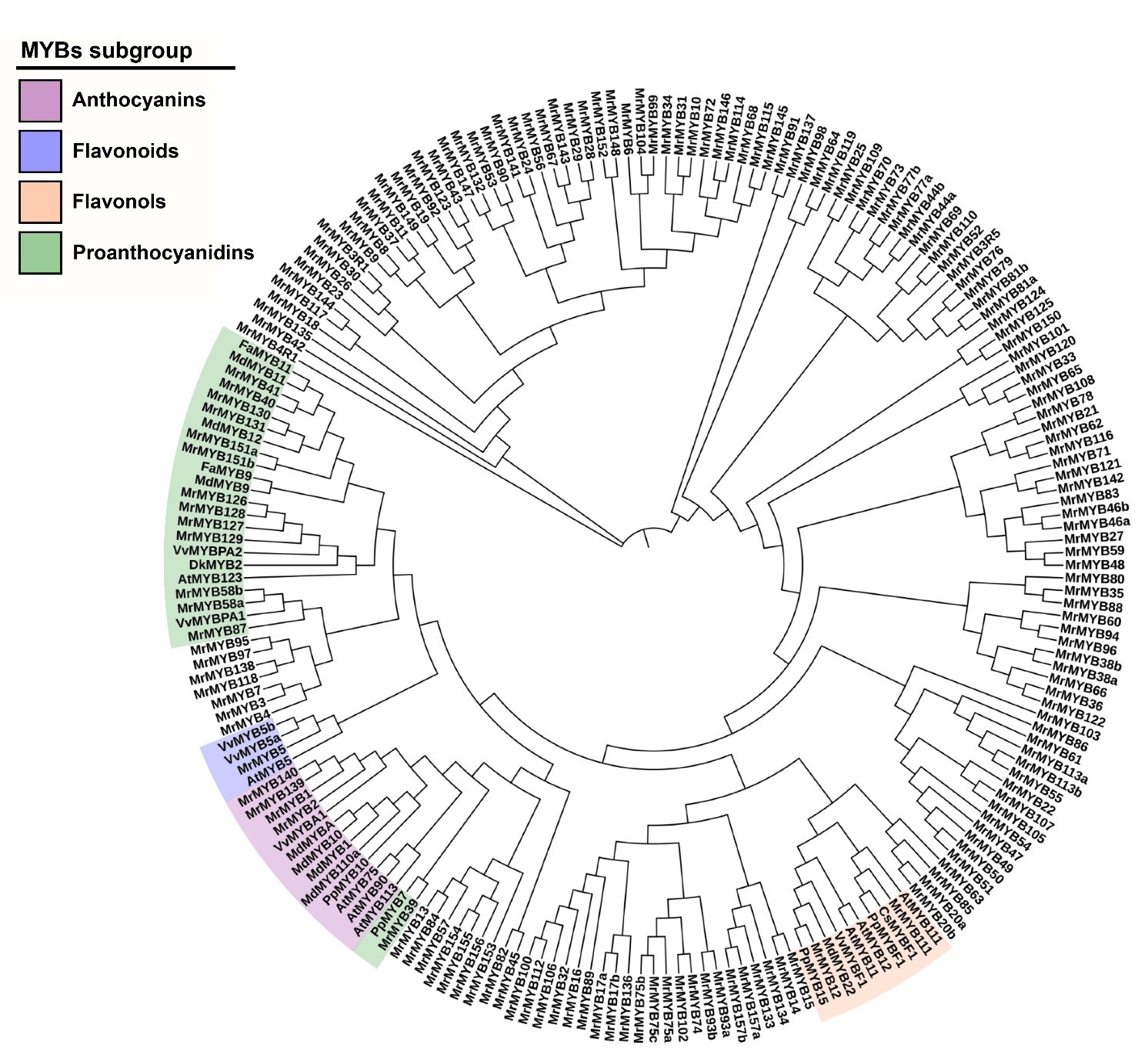

Supplement: Supplementary Figure 1 — The sequence logos of the R2 (A) and R3 (B) MYB repeats. These logos were based on the multiple sequence alignment of 122 R2R3-MYBs in Chinese bayberry. The core bits indicate the information content for each position in the sequence. The asterisks indicate the typical conserved Trp residues in the MYB domain. The triangle indicates the residue in the R3 repeat with positions identical to the first conserved Trp residue in the R2 repeat. [file Data_Sheet_1.docx]
